# The UbiB family member Cqd1 forms a novel membrane contact site in mitochondria

**DOI:** 10.1242/jcs.260578

**Published:** 2023-05-18

**Authors:** Siavash Khosravi, Xenia Chelius, Ann-Katrin Unger, Daniela Rieger, Johanna Frickel, Timo Sachsenheimer, Christian Lüchtenborg, Rico Schieweck, Britta Brügger, Benedikt Westermann, Till Klecker, Walter Neupert, Max E. Harner

**Affiliations:** ^1^ Department of Cell Biology, Biomedical Center, Medical Faculty, Ludwig-Maximilians University Munich, 82152 Planegg/Martinsried, Germany; ^2^ Institute of Cell Biology, University of Bayreuth, 95440 Bayreuth, Germany; ^3^ Heidelberg University Biochemistry Center (BZH), 69120 Heidelberg, Germany

**Keywords:** Mitochondria, Contact sites, UbiB protein family, Phospholipids, Mitochondrial biogenesis, Mitochondrial morphology

## Abstract

Mitochondria are essential organelles of eukaryotic cells and are characterized by their unique and complex membrane system. They are confined from the cytosol by an envelope consisting of two membranes. Signals, metabolites, proteins and lipids have to be transferred across these membranes via proteinaceous contact sites to keep mitochondria functional. In the present study, we identified a novel mitochondrial contact site in *Saccharomyces cerevisiae* that is formed by the inner membrane protein Cqd1 and the outer membrane proteins Por1 and Om14. Similar to what is found for the mitochondrial porin Por1, Cqd1 is highly conserved, suggesting that this complex is conserved in form and function from yeast to human. Cqd1 is a member of the UbiB protein kinase-like family (also called aarF domain-containing kinases). It was recently shown that Cqd1, in cooperation with Cqd2, controls the cellular distribution of coenzyme Q by a yet unknown mechanism. Our data suggest that Cqd1 is additionally involved in phospholipid homeostasis. Moreover, overexpression of *CQD1* and *CQD2* causes tethering of mitochondria to the endoplasmic reticulum, which might explain the ability of Cqd2 to rescue ERMES deletion phenotypes.

## Teaser

**Summary:** The conserved yeast mitochondrial inner membrane protein Cqd1 interacts with the outer membrane proteins Por1 and Om14. Additionally, Cqd1 is important for maintaining mitochondrial homeostasis.

## INTRODUCTION

Mitochondria are essential organelles in eukaryotic cells and are characterized by an envelope consisting of two membranes. In addition to their important role in the cellular energy metabolism, mitochondria perform a multitude of other functions, including the synthesis of proteins, iron-sulfur clusters and lipids. Specialized structures in the mitochondrial membranes, so-called contact sites, have to be present to enable mitochondria to perform all these functions. The term contact site often refers to inter-organellar contacts, for instance between mitochondria and the endoplasmic reticulum (ER) (Kornmann et al., 2009; Murley et al., 2015), mitochondria and vacuoles, the yeast equivalent of mammalian lysosomes (Gonzalez Montoro et al., 2018; John Peter et al., 2017), or mitochondria and lipid droplets (Pu et al., 2011). In addition, intra-mitochondrial contact sites between the mitochondrial inner and outer membrane can be observed (Tamura et al., 2019). Importantly, both kinds of contacts are crucial for mitochondrial functions (Khosravi and Harner, 2020; Klecker et al., 2014; Kornmann, 2020; Phillips and Voeltz, 2016; Renne and Hariri, 2021).

Most contacts between the mitochondrial inner and outer membrane depend on the mitochondrial contact site and cristae-organizing system (MICOS), a multi-subunit protein complex in the mitochondrial inner membrane (Harner et al., 2011; Hoppins et al., 2011; von der Malsburg et al., 2011). This highly conserved complex interacts with at least six different proteins or complexes of the outer membrane – the Fzo1–Ugo1 complex (Harner et al., 2011), the Miro GTPases (Modi et al., 2019), Om45 (Hoppins et al., 2011), Por1 (Hoppins et al., 2011), the TOB (SAM) complex (Bohnert et al., 2012; Darshi et al., 2011; Harner et al., 2011; Körner et al., 2012; Xie et al., 2007; Zerbes et al., 2012) and the TOM complex (Bohnert et al., 2012; von der Malsburg et al., 2011; Zerbes et al., 2012).

In addition, several other intra-mitochondrial contact sites exist that do not depend on MICOS. Some of these have been known for decades, such as the TIM23–TOM super complex, which is transiently formed during protein import and can be stabilized by the arrest of precursor proteins (Chacinska et al., 2003; Dekker et al., 1997; Schleyer and Neupert, 1985; Schwaiger et al., 1987), or the mitochondrial fusion machineries (Fritz et al., 2001; Sesaki et al., 2003; Wong et al., 2003). Others were identified just recently, for instance the contact site formed by Por1 (yeast porin) and Mdm31, which has been suggested to be important for the biosynthesis of cardiolipin (Miyata et al., 2018). Apparently, the functions of these intra-mitochondrial contacts are quite diverse, varying from protein import to lipid transport and the formation of mitochondrial architecture and morphology.

Members of the poorly characterized UbiB protein kinase-like family (aarF domain-containing kinases) were recently implicated in mitochondrial membrane homeostasis (Awad et al., 2020; Johnson et al., 2005; Kemmerer et al., 2021; Mollet et al., 2008; Odendall et al., 2019; Reidenbach et al., 2018; Stefely et al., 2015). UbiB family members are defined by the presence of a protein kinase-like domain (PKL) of unknown function. *Saccharomyces cerevisiae* has three family members which all reside in mitochondria – Coq8 (Abc1), Cqd1 (Mco76) and Cqd2 (Mcp2) (Do et al., 2001; Morgenstern et al., 2017; Tan et al., 2013). The bacterial proteins UbiB (*Escherichia coli*) and AarF (*Providencia stuartii*) are essential for the synthesis of coenzyme Q (ubiquinone) (Macinga et al., 1998; Poon et al., 2000). Likewise, Coq8 and its mammalian homolog ADCK3 are also part of the coenzyme Q biosynthesis pathway (Do et al., 2001; Johnson et al., 2005; Mollet et al., 2008; Reidenbach et al., 2018; Stefely et al., 2015). While our work was in progress, an elegant study linked also Cqd1 and Cqd2 to coenzyme Q homeostasis. Deletion of *CQD1* results in excess export of coenzyme Q to extra-mitochondrial membranes, whereas deletion of *CQD2* leads to an accumulation of excess coenzyme Q in mitochondria. Thus, Cqd1 and Cqd2 apparently perform antagonistic roles in the distribution of coenzyme Q between mitochondria and other organelles (Kemmerer et al., 2021).

The ER–mitochondria encounter structure (ERMES) establishes physical contacts between the mitochondrial outer membrane and the ER (Klecker et al., 2014; Kornmann et al., 2009). Interestingly, it has been shown that overexpression of *CQD2* (alternative name *MCP2*) can rescue the phenotypes caused by the deletion of ERMES subunits (Tan et al., 2013). However, the molecular mechanism is unknown.

In the present study, we set out to functionally analyze the conserved mitochondrial protein encoded by the ORF *YPL109C*, which is now named Cqd1. We identified a MICOS-independent contact site formed by Cqd1 in the mitochondrial inner membrane and Por1 and Om14 in the outer membrane. Our characterization of the Δ*cqd1* deletion mutant suggests that Cqd1 contributes to phospholipid homeostasis in addition to its role in the regulation of the distribution of coenzyme Q. Moreover, we obtained evidence that defined levels of UbiB family members are important for mitochondrial morphology and architecture. We find that overexpression of these mitochondrial inner membrane proteins results in their integration into the outer membrane and tethering of mitochondria to the ER. This might explain the compensatory effect of Cqd2 for ERMES deletion mutants (Tan et al., 2013).

## RESULTS

### Cqd1 is a mitochondrial inner membrane protein facing the intermembrane space

**Fig. 1.**
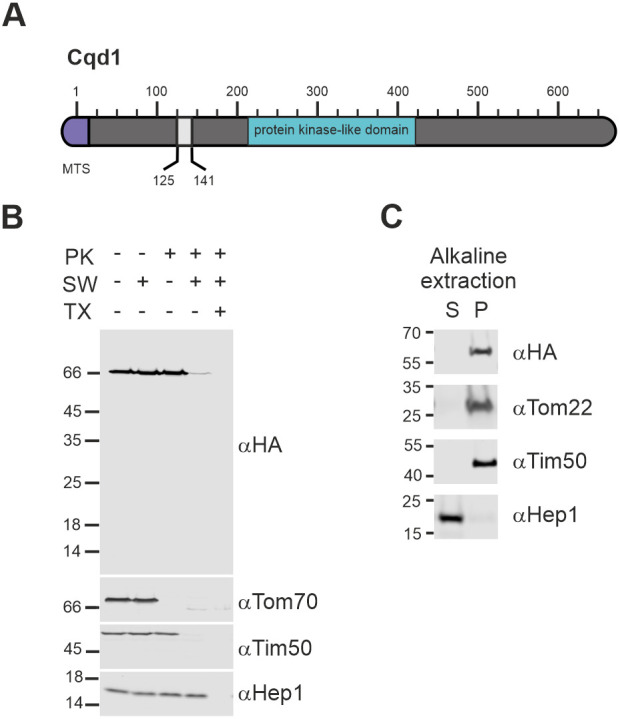
**Cqd1 is a mitochondrial inner membrane protein exposing its C-terminus to the intermembrane space.** (A) Schematic representation of Cqd1. Violet, mitochondrial targeting sequence (MTS; amino acids 1–15); grey, predicted transmembrane domain (amino acids 125–141); turquoise, conserved protein kinase-like domain (amino acids 213–421). (B) Cqd1 exposes its C-terminus to the intermembrane space. Mitochondria isolated from a Cqd1–3xHA-expressing strain were treated with isotonic buffer, subjected to swelling by incubation in hypotonic buffer to disrupt the outer membrane (SW), or lysed using a Triton X-100 containing buffer (TX). Proteinase K (PK) was added as indicated. Samples were analyzed by SDS-PAGE and immunoblotting. Tom70 was used as a marker for the outer membrane, Tim50 for the inner membrane and Hep1 for the matrix. (C) Cqd1 is an integral membrane protein. Isolated mitochondria were subjected to alkaline extraction to separate soluble and membrane proteins. Soluble proteins present in the supernatant (S) and membrane proteins in the pellet (P) were analyzed by SDS-PAGE and immunoblotting. Blots are representative of at least three repeats.

We first asked where Cqd1 is precisely located within mitochondria. Computational analysis using MitoProt II (Claros and Vincens, 1996) revealed that Cqd1 contains a potential N-terminal mitochondrial-targeting sequence (MTS; amino acids 1–15) (Fig. 1A). In addition, a predicted transmembrane segment (amino acids 125–141) can be identified (Fig. 1A) (SACS MEMSAT2 transmembrane prediction; Jones et al., 1994). We used an epitope-tagged construct (Cqd1–3xHA) to analyze the topology of Cqd1. Proteinase K (PK) accessibility and alkaline extraction assays using isolated mitochondria showed that it is behaving like Tim50, which is anchored in the inner membrane with an α-helical transmembrane domain (Fig. 1B,C). PK did not degrade Cqd1–3xHA in intact mitochondria, but the signal was largely gone when PK was added to hypotonically swollen mitochondria with a disrupted outer membrane. No shorter fragment appeared upon addition of PK to swollen mitochondria indicating that the C-terminal tag was degraded (Fig. 1B). Furthermore, Cqd1–3xHA remained in the pellet fraction upon alkaline extraction, like the membrane proteins Tom22 and Tim50 (Fig. 1C). It should be noted that in western blot analyses Cqd1–3xHA shows an apparent size of 66 kDa, instead of its calculated molecular mass of 76 kDa (untagged full-length Cqd1) or 74 kDa (untagged Cqd1 minus the MTS) (Fig. 1B). We conclude that Cqd1 is present in the mitochondrial inner membrane with its major C-terminal part facing the intermembrane space.

### The *CQD1* gene negatively interacts with *UPS1* and *CRD1*

**Fig. 2.**
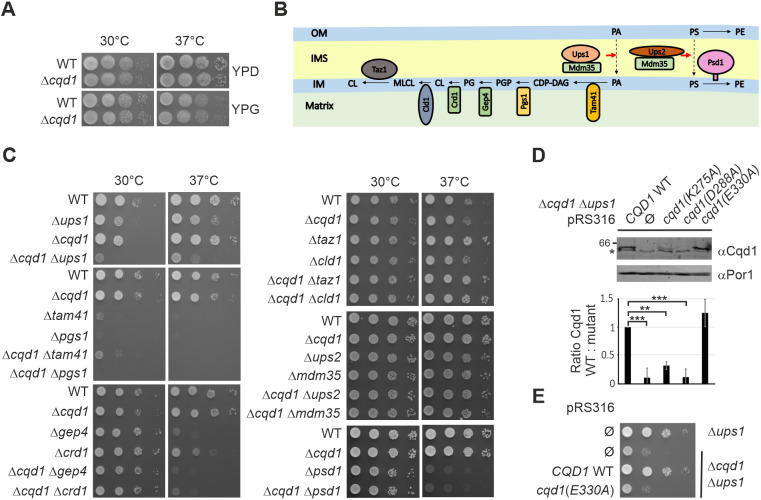
***CQD1* is a negative genetic interactor of *UPS1* and *CRD1*.** (A) Deletion of *CQD1* does not result in a growth defect. Cells of wild-type (WT) and a Δ*cqd1* deletion strain were grown to logarithmic growth phase in rich medium containing glucose as carbon source (YPD). Cell growth was analyzed by drop dilution assay on plates containing rich medium supplemented with either glucose (YPD) or glycerol (YPG) at 30°C or 37°C. (B) Schematic illustration of the mitochondrial phospholipid metabolism. OM, outer membrane; IMS, intermembrane space; IM, inner membrane; PA, phosphatidic acid; CDP-DAG, cytidine diphosphate diacylglycerol; PGP, phosphatidylglycerol phosphate; PG, phosphatidylglycerol; CL, cardiolipin; MLCL, monolysocardiolipin; PS, phosphatidylserine; PE, phosphatidylethanolamine. (C) Deletion of *CQD1* from cells lacking Ups1 or Crd1 results in a synthetic growth defect. Cells were treated as in A with the difference that they were shifted to synthetic medium containing glucose (SCD) 30 h before growth analysis on SCD plates. (D) The conserved amino acids K275 and D288 in the predicted protein kinase-like domain are important for the stability of Cqd1. Cells bearing either the empty plasmid (Ø) or plasmids carrying the respective *cqd1* alleles were grown in SCGal. Crude mitochondria were isolated and the Cqd1 levels were analyzed by immunoblotting using an anti-Cqd1 antibody. Upper panel, immunoblot of one representative experiment. The asterisk indicates a cross reaction of the anti-Cqd1 antibody. Lower panel, quantitative analysis of Cqd1 steady-state level in the different strains analyzed in three biological replicates. Quantification was undertaken with Image Studio software. Error bars indicate s.d. **P≤0.01; ***P≤0.001 (one-way ANOVA with subsequent Tukey's multiple comparison test). (E) Glutamic acid 330 is essential for the function of Cqd1. Cells bearing either the empty plasmid or a plasmid carrying *CQD1* wild type (WT) or *cqd1(E330A)* alleles were treated as in C. Images in A, C and E are representative of at least three repeats.

Several high-throughput screens have shown that cells lacking Cqd1 are able to grow on non-fermentable carbon sources (Qian et al., 2012; Stenger et al., 2020). Also, Kemmerer et al. (2021) observed growth of Δ*cqd1* cells under respiratory conditions in liquid medium, although growth was reduced when coenzyme Q precursors were depleted. We generated a deletion mutant lacking Cqd1 and analyzed its growth phenotype under several conditions on agar plates. We observed that the Δ*cqd1* mutant grows to the same degree as wild-type on fermentable and non-fermentable carbon sources at normal and elevated temperatures (Fig. 2A) confirming that Cqd1 is not essential for respiratory growth.

Mitochondria are intensively involved in the cellular phospholipid metabolism as they generate cardiolipin and phosphatidylethanolamine. Interestingly, two independent high-throughput studies revealed a genetic interaction of *CQD1* with *UPS1* (Costanzo et al., 2016; Hoppins et al., 2011). Ups1 is responsible for transport of phosphatidic acid across the intermembrane space (Fig. 2B) (Connerth et al., 2012; Tamura et al., 2012). Thus, we generated double deletion mutants lacking *CQD1* and genes encoding proteins involved in mitochondrial phospholipid metabolism, including *CLD1*, *CRD1*, *GEP4*, *MDM35*, *PGS1*, *PSD1*, *TAM41*, *TAZ1*, *UPS1* and *UPS2* (Fig. 2B). Growth analysis revealed a strong defect for the double deletion mutant Δ*cqd1* Δ*ups1* specifically on synthetic medium (Fig. 2C). A weaker, yet reproducible, growth defect at 37°C could be detected for the double deletion mutant Δ*cqd1* Δ*crd1*. All other double deletion mutants did not show an enhanced growth phenotype. The growth defect of the single deletion mutants Δ*tam41* and Δ*pgs1* was so strong that we cannot judge whether the additional deletion of *CQD1* further reduces cell growth (Fig. 2C).

Cqd1 is a member of the highly conserved UbiB family (Kemmerer et al., 2021; Stefely et al., 2015) and thus shows a predicted protein kinase-like domain (amino acids 213–421, Saccharomyces Genome Database, SGD; Cherry et al., 2012) (Fig. 1A). Therefore, we asked whether the protein kinase-like domain of Cqd1 is important for its function. We generated mutants in conserved amino acid residues within this domain (K275A, D288A and E330A) to inhibit ATP binding, similar to what was undertaken previously in studies on Cqd2 and Coq8 (Odendall et al., 2019; Reidenbach et al., 2018; Stefely et al., 2015). Of note, introduction of the point mutations K275A or D288A led to strongly reduced steady state levels of Cqd1 indicating reduced expression or stability of the protein (Fig. 2D). Therefore, we did not further analyze these alleles. In contrast, the introduction of the point mutation E330A did not affect the steady state level of Cqd1 (Fig. 2D). Whereas expression of wild-type Cqd1 largely restored the growth of the double deletion mutant Δ*cqd1* Δ*ups1*, the *cqd1(E330A)* allele did not, indicating that ATP binding of Cqd1 is important for its function (Fig. 2E). These results are in accordance with observations recently made by Kemmerer et al. (2021) who reported that alleles carrying mutations of conserved residues of the kinase-like domain were unable to rescue Δ*cqd1* growth defects.

In summary, we could manually confirm the negative genetic interaction of *CQD1* and *UPS1*, and identified *CRD1* as a novel negative interactor of *CQD1*. Interestingly, both genetic interactors are involved in biosynthesis of the phospholipid cardiolipin. Furthermore, Cqd1 activity depends on an intact protein kinase-like domain.

### Cqd1 contributes to mitochondrial lipid homeostasis

**Fig. 3.**
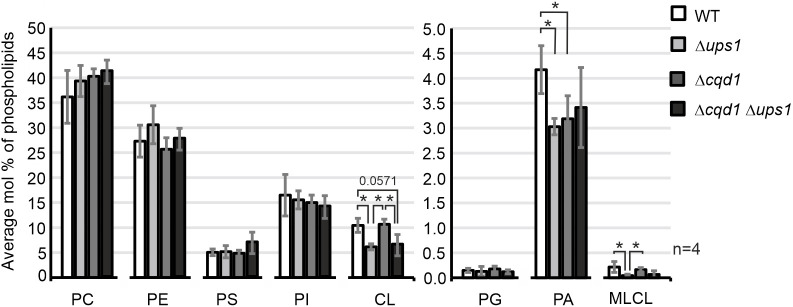
**Cqd1 is involved in mitochondrial phospholipid homeostasis.** Strains were grown in synthetic medium containing glycerol (SCG) and mitochondria were purified by sucrose gradient centrifugation. Phospholipids were extracted and analyzed by mass spectrometry. The level of each phospholipid species (percentage of total phospholipids) is shown as a mean±s.d. of four biological replicates. *P≤0.05 (Mann–Whitney test). PC, phosphatidylcholine; PE, phosphatidylethanolamine; PS, phosphatidylserine; PI, phosphatidylinositol; CL, cardiolipin; PG, phosphatidylglycerol; PA, phosphatidic acid; MLCL, monolysocardiolipin. Relative amounts of different species of single phospholipids are presented in Table S2.

Next, we analyzed the phospholipid composition of mitochondria isolated from wild-type yeast, the Δ*ups1* or Δ*cqd1* single deletion strains and the Δ*cqd1* Δ*ups1* double deletion mutant by mass spectrometry. Consistent with previous results (Connerth et al., 2012), deletion of *UPS1* resulted in a severe reduction of cardiolipin and monolysocardiolipin (Fig. 3; Table S1). Also, we observed a significant reduction of phosphatidic acid in Δ*ups1*, which might depend on growth conditions (Eiyama et al., 2021). Interestingly, the lipidomics analysis of mitochondria obtained from Δ*cqd1* revealed a significant reduction of phosphatidic acid, whereas the levels of the other phospholipids remained unchanged (Fig. 3; Table S1). The double deletion mutant Δ*cqd1* Δ*ups1* did not show a synthetic phenotype. We suggest that Cqd1 is involved not only in maintenance of mitochondrial coenzyme Q levels (Kemmerer et al., 2021), but also modulates the levels of phosphatidic acid (Fig. 3, Table S1).

### Mitochondrial biogenesis and dynamics are impaired in the Δ*cqd1* Δ*ups1* double mutant

**Fig. 4.**
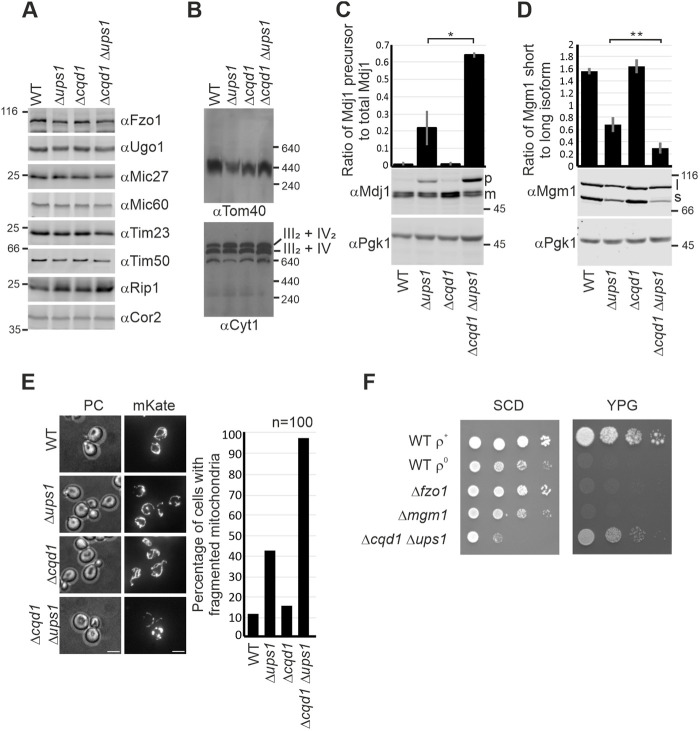
**Simultaneous deletion of *CQD1* and *UPS1* impairs mitochondrial protein import and dynamics.** (A) Analysis of steady-state levels of mitochondrial proteins in wild-type (WT) cells and cells lacking Ups1, Cqd1, or both. Cells were grown in synthetic complete medium containing galactose (SCGal), and whole-cell extracts were analyzed by immunoblotting. (B) Formation of mitochondrial protein complexes. Strains were grown in SCG. Isolated mitochondria were lysed in digitonin-containing buffer (3% w/v) and cleared lysates were subjected to BN-PAGE. The assembly of the TOM complex and respiratory chain super complexes were analyzed by immunoblotting using antibodies against Tom40 or Cyt1. (C) Deletion of *CQD1* in cells lacking Ups1 exacerbates accumulation of the precursor of Mdj1. Cells were grown in SCD, whole-cell lysates were prepared and analyzed by immunoblotting with specific antibodies. Pgk1 served as a loading control. p, precursor of Mdj1; m, mature form of Mdj1. The quantification was obtained from three independent experiments and shows mean±s.d. of the ratio of the Mdj1 precursor to the total amount of Mdj1. Quantification was undertaken with Image Studio software. *P≤0.05 (unpaired two-tailed Student's *t*-test). (D) Simultaneous deletion of *CQD1* and *UPS1* leads to strongly reduced processing of Mgm1. Whole-cell lysates from cells grown in SCD were analyzed by immunoblotting. l, long isoform of Mgm1; s, short isoform of Mgm1. The quantification was obtained from three independent experiments and shows mean±s.d. of the ratio of the short form to the long form of Mgm1. Quantification was undertaken with Image Studio software. **P≤0.01 (unpaired two-tailed Student's *t*-test). (E) Mitochondria in cells lacking Ups1 and Cqd1 are highly fragmented. Mitochondria were labeled by expression of mKate targeted to mitochondria. Cells were grown in YPD and shifted to SCD, harvested in their logarithmic growth phase and immobilized on slides covered with concanavalin A. PC, phase contrast. For quantification 100 cells were counted for each strain. Scale bars: 4 µm. (F) The synthetic growth defect of the double deletion mutant Δ*cqd1* Δ*ups1* is not caused by loss of mitochondrial DNA. Cells of the indicated strains were grown to logarithmic phase on YPD, shifted to SCD and growth was analyzed by drop dilution assay on SCD and YPG plates at 30°C. Images in A, B and F are representative of at least three repeats.

The drastic growth phenotype of the Δ*cqd1* Δ*ups1* double mutant suggests that this strain struggles with severe problems in essential mitochondrial functions, which might be caused by a lack of mitochondrial proteins or complexes involved in mitochondrial dynamics, architecture, protein import and/or respiration. Therefore, we analyzed the steady state levels of various proteins involved in these processes, including Fzo1, Ugo1, Mic27, Mic60, Tim23, Tim50, Rip1 and Cor2. Also, we tested the assembly of the TOM complex and the respiratory chain super complexes by native gel electrophoresis. However, a comparison of the Δ*cqd1* Δ*ups1* double mutant with wild-type and the single deletion mutants revealed no significant defects (Fig. 4A,B).

Previous studies have shown that deletion of *UPS1* compromises two processes that both depend on the mitochondrial membrane potential. First, the import of the mitochondrial matrix-localized chaperone Mdj1 is impaired, resulting in the accumulation of unprocessed precursor (Tamura et al., 2009). And second, processing of Mgm1, a large inner membrane-associated GTPase required for mitochondrial fusion, is reduced, resulting in the accumulation of the long isoform, l-Mgm1 (Sesaki et al., 2006). Interestingly, deletion of *CQD1* neither affected the import of Mdj1 nor processing of Mgm1. However, we found that both the defects observed in the Δ*ups1* single mutant were exacerbated in the Δ*cqd1* Δ*ups1* double mutant. We observed a strong accumulation of Mdj1 precursor protein and an almost complete inhibition of Mgm1 processing (Fig. 4C,D). Previous studies have shown that a balanced ratio of processed s-Mgm1 to unprocessed l-Mgm1 is necessary for mitochondrial fusion (DeVay et al., 2009; Zick et al., 2009). Accordingly, we observed a wild-type-like mitochondrial network in the Δ*cqd1* single deletion mutant. In the Δ*cqd1* Δ*ups1* double deletion mutant, however, the mitochondrial network appeared almost completely fragmented, indicating impairment of mitochondrial fusion (Fig. 4E). Mitochondrial fragmentation leads to the loss of mitochondrial DNA (mtDNA), as shown for Δ*mgm1* or Δ*fzo1* deletion mutants (Hermann et al., 1998; Rapaport et al., 1998; Sesaki et al., 2003). Therefore, we next tested whether the growth phenotype observed in the Δ*cqd1* Δ*ups1* double mutant (Fig. 2C) might be due to the lack of mtDNA. Growth analysis of wild-type cells with (ρ^+^) and without mtDNA (ρ^0^), Δ*fzo1* cells, Δ*mgm1* cells and Δ*cqd1* Δ*ups1* cells revealed that loss of mtDNA is not the reason for the strong phenotype on SCD medium. Additionally, the Δ*cqd1* Δ*ups1* double mutant was able to grow on respiratory medium, indicating that this strain contains functional mtDNA. The reduced growth on YPG indicated, however, that this strain starts to lose respiratory competence (Fig. 4F).

Thus, the relatively mild phenotypes of the Δ*ups1* single deletion mutant regarding cell growth, generation of membrane potential and morphology of mitochondria (Sesaki et al., 2006; Tamura et al., 2009) are enhanced by additional deletion of the *CQD1* gene.

### Cqd1 and Cqd2 have antagonistic roles in mitochondrial biogenesis

**Fig. 5.**
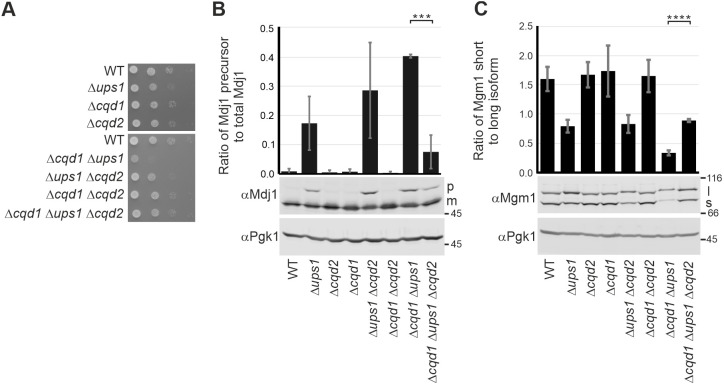
**Deletion of *CQD2* restores the phenotypes of a Δ*cqd1* Δ*ups1* double deletion.** (A) Deletion of *CQD2* in the Δ*cqd1* Δ*ups1* background rescues the growth phenotype. Strains were grown to logarithmic phase and growth was analyzed by drop dilution assay on SCD plates at 30°C. (B,C) Mdj1 import and Mgm1 processing are restored in the Δ*cqd1* Δ*ups1* Δ*cqd2* triple mutant. Cells were grown in SCD, whole-cell lysates were prepared and analyzed by immunoblotting. Pgk1 served as a loading control. (B) p, precursor of Mdj1; m, mature form of Mdj1. The quantification (Image Studio software) was obtained from three independent experiments and shows means of the ratio of the Mdj1 precursor to the total amount of Mdj1. (C) l, long isoform of Mgm1; s, short isoform of Mgm1. The quantification (Image Studio software) was obtained from three independent experiments and shows means of the ratio of the short form to the long form of Mgm1. Error bars indicate s.d. ***P≤0.001; ****P≤0.0001 (unpaired two-tailed Student's *t*-test). Images in A are representative of at least three repeats.

A previous study indicated opposing functions of Cqd1 and Cqd2 (Kemmerer et al., 2021). Therefore, we generated all possible single, double and triple deletion mutants to analyze the interaction network of *CQD1*, *CQD2* and *UPS1*. Strikingly, growth analysis of the triple deletion mutant Δ*cqd1* Δ*ups1* Δ*cqd2* revealed that deletion of *CQD2* in the Δ*cqd1* Δ*ups1* background completely rescued the growth phenotype (Fig. 5A). At the same time, the accumulation of Mdj1 precursor protein and the impaired Mgm1 processing were largely rescued (Fig. 5B,C). This suggests that the equilibrium of the putative kinases Cqd1 and Cqd2 is particularly important when mitochondrial membrane lipid composition is disturbed through deletion of *UPS1*.

### Cqd1 is part of a MICOS-independent contact site

**Fig. 6.**
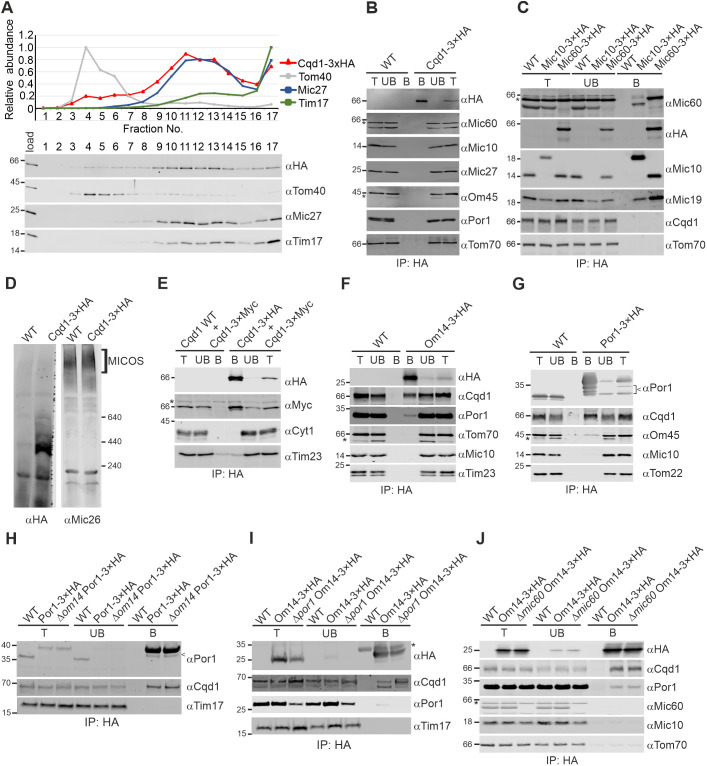
**Cqd1 forms a novel contact site with Om14 and Por1.** (A) Cqd1 is enriched in contact site fractions. Mitochondria from a Cqd1–3xHA-expressing strain were isolated, subjected to osmotic treatment, sonication and sucrose density gradient centrifugation. The gradient was fractionated, proteins were subjected to TCA precipitation and analyzed by immunoblotting. Top, the graph shows mean values of three independent experiments for the distribution of Cqd1–3xHA and the marker proteins for the outer membrane (Tom40), the inner membrane (Tim17) or contact sites (Mic27). Error bars indicating s.d. are shown in Fig. S1A. Bottom, immunoblot from one representative experiment. Load, 10% of material applied to gradient. (B) Mic10 and Mic60 do not co-precipitate with Cqd1. Mitochondria of wild-type (WT) and a yeast strain expressing Cqd1–3xHA were isolated and lysed in digitonin-containing buffer (1% w/v). Lysates were subjected to immunoprecipitation using anti-HA affinity agarose. The indicated fractions were analyzed by SDS-PAGE and immunoblotting. T, total lysate (5%); UB, unbound protein (5%); B, bound protein (100%). Asterisks indicate cross reactions of the antibodies against Mic60 or Om45. (C) Cqd1 does not co-precipitate with Mic10 or Mic60. Mitochondria of wild-type or yeast strains expressing Mic10–3xHA or Mic60–3xHA were analyzed as in B. T, total lysate (2.5%); UB, unbound protein (2.5%); B, bound protein (100%). The asterisk indicates a cross reaction of the anti-Mic60 antibody. As the cross reaction of the Mic60 antibody shows the same size as Mic60–3xHA, an immunodecoration of this membrane fragment with an anti-HA antibody is presented additionally. (D) Cqd1 forms high molecular mass complexes. Mitochondria isolated from wild-type and a yeast strain expressing Cqd1–3xHA were solubilized in digitonin (3% w/v). Cleared lysates were subjected to BN-PAGE. Cqd1–3xHA-containing complexes were detected by immunoblotting with an anti-HA antibody. Analysis of the MICOS complex using an anti-Mic26 antibody served as control. (E) Cqd1 interacts homotypically. Mitochondria of yeast strains expressing Cqd1–3xMyc in the presence of untagged or 3xHA-tagged Cqd1 were treated as described in B. T, total lysate (5%); UB, unbound protein (5%); B, bound protein (100%). The asterisk indicates a cross reaction of the anti-Myc antibody. (F) Cqd1 interacts with Om14. Mitochondria of wild-type and a yeast strain expressing Om14–3xHA were analyzed as in B. T, total lysate (2.5%); UB, unbound protein (2.5%); B, bound protein (100%). The asterisk indicates a cross reaction of the anti-Tom70 antibody. (G) Cqd1 interacts with Por1. Mitochondria of wild-type or a yeast strain expressing Por1–3xHA were analyzed as in B. T, total lysate (1%); UB, unbound protein (1%); B, bound protein (100%). The arrowhead indicates degradation products of Por1–3xHA. The asterisk indicates a cross reaction of the anti-Om45 antibody. (H) The Cqd1–Por1 interaction is independent of Om14. Mitochondria of wild-type cells, Por1–3xHA cells and Δ*om14* Por1–3xHA cells were analyzed as in G. Knockout was confirmed by PCR. The arrowhead indicates a degradation product of Por1–3xHA. (I) The Cqd1–Om14 interaction does not depend on Por1. Mitochondria of wild-type cells, Om14–3xHA cells and Δ*por1* Om14–3xHA cells were analyzed as in F. Knockout was confirmed by PCR. The band detectable in Δ*por1* Om14–3xHA (T and UB fractions) using the anti-Por1 antibody is probably a cross reaction with its paralog Por2. The asterisk indicates IgGs. (J) The contact site formed by Cqd1 and Om14 is independent of MICOS. Mitochondria of wild-type, Om14–3×HA and Δ*mic60* Om14–3×HA strains were analyzed as in F. The asterisk indicates a cross reaction of the anti-Mic60 antibody. Images in B–J are representative of at least three repeats.

Our results described above, and the observations reported by Kemmerer et al. (2021) suggest that Cqd1 is involved in mitochondrial lipid homeostasis. We reasoned that a membrane contact site would be ideally suited for such a function. To test whether Cqd1 is a contact site protein, we used an established method to fractionate mitochondria, which is based on sonication, dounce homogenization and subsequent density gradient centrifugation of mitochondrial particles. Owing to the physical properties of the mitochondrial outer and inner membranes, outer membrane proteins will be enriched in low-density fractions at the top of the gradient and inner membrane proteins will accumulate in high-density fractions at the bottom of the gradient. Inner membrane proteins that qualify for contact site proteins are shifted to fractions of intermediate density through their interaction with the outer membrane (Harner et al., 2011). Strikingly, western blot analysis of mitochondrial fractions revealed that the distribution of Cqd1 is clearly distinguishable from both the outer membrane marker Tom40 and the inner membrane marker Tim17. Cqd1 was significantly enriched in fractions of intermediate density (fraction numbers 10–14), very similar to what is seen for the MICOS subunit Mic27 (Fig. 6A; Fig. S1A).

In order to analyze whether Cqd1 is a novel MICOS subunit, we performed immunoprecipitation of Cqd1 and different MICOS subunits. Neither the isolation of Cqd1 resulted in co-isolation of the MICOS subunits Mic10 or Mic60 nor vice versa (Fig. 6B,C). Native gel electrophoresis revealed that Cqd1 is present in a high molecular mass complex of ∼400 kDa, a size that is clearly different from the 1.5 MDa large MICOS complex (Fig. 6D). These results suggest that Cqd1 is part of a novel contact site rather than a new MICOS subunit.

To screen for possible interaction partners of Cqd1, we first tested whether it might interact homotypically. To this end, a yeast strain was generated expressing simultaneously a 3xHA-tagged and a 3xMyc-tagged version of Cqd1. Immunoprecipitation of Cqd1–3xHA led to efficient co-isolation of Cqd1–3xMyc, revealing homotypic interactions of Cqd1 (Fig. 6E).

Although the homotypic Cqd1 interaction could explain the formation of the high molecular mass complex (Fig. 6D), it does not explain the presence of Cqd1 in contact site fractions (Fig. 6A). Therefore, chemical crosslinking was used to get an indication of further interaction partners. Although incubation with di(N-succinimidyl) glutarate (DSG) did not reveal any crosslink partners, treatment with m-maleimidobenzoyl-N-hydoxysuccinimide ester (MBS) produced two prominent crosslinks (Fig. S1B).

These two crosslink products of ∼80 and ∼100 kDa indicated interaction partners with a molecular mass of ∼15 and ∼30 kDa. As this experiment did not reveal the identity of the crosslink partners of Cqd1, we directly tested the interaction of Cqd1 with outer membrane proteins of appropriate size by co-immunoprecipitation. Om14 and Por1 are two highly abundant proteins forming a complex in the mitochondrial outer membrane (Lauffer et al., 2012; Sakaue et al., 2019). We found that both proteins are in the same complex with Cqd1 (Fig. 6F,G). The co-isolation efficiency of Cqd1 with Por1 was even higher than the co-isolation efficiency of Om45, a previously identified interaction partner of Por1 in the outer membrane (Lauffer et al., 2012; Sakaue et al., 2019; Wenz et al., 2014) (Fig. 6G). Of note, we were not able to co-purify Por1 by immunoprecipitating Cqd1–3xHA (Fig. 6B), indicating that immunoprecipitation of Cqd1 impacts the integrity of the complex. Interestingly, Por1 was recently shown to form a contact site with the mitochondrial inner membrane protein Mdm31 (Miyata et al., 2018). Therefore, it might be that Cqd1 is a novel subunit of this complex. However, immunoprecipitating Mdm31–3xHA did not result in co-isolation of Cqd1 (our unpublished observations).

Next, we asked whether Om14 or Por1 is the direct interaction partner of Cqd1. Interestingly, neither deletion of *OM14* nor of *POR1* affected the capability to co-immunoprecipitate Cqd1 with Por1–3xHA or Om14–3xHA, respectively (Fig. 6H,I). This suggests that Cqd1 interacts with both proteins directly, possibly in an independent manner. Of note, although we deleted *POR1* in the Om14–3xHA-expressing strain, we still obtained a weak signal upon immunodecoration (Fig. 6I), probably reflecting a cross reaction with its paralog Por2.

Most of the contact sites between the mitochondrial inner and outer membranes identified to date depend on MICOS (Bohnert et al., 2012; Darshi et al., 2011; Harner et al., 2011; Hoppins et al., 2011; Körner et al., 2012; Modi et al., 2019; von der Malsburg et al., 2011; Xie et al., 2007; Zerbes et al., 2012). In particular, Mic60 is essential for the integrity of the MICOS complex as well as the formation of MICOS-dependent contact sites (Harner et al., 2011; Hoppins et al., 2011; von der Malsburg et al., 2011). Although we did not detect a physical interaction between Cqd1 and MICOS, the interaction of Cqd1 with Por1 and Om14 might still depend on the presence of an intact MICOS complex. To test this, we performed immunoprecipitation of Om14–3xHA expressed in the wild-type background or the Δ*mic60* deletion background. We observed that immunoprecipitation of Om14–3xHA led to the successful co-isolation of Cqd1 and Por1 independently of the presence of Mic60 (Fig. 6J).

Taken together, we identified a novel contact site formed by the mitochondrial inner membrane protein Cqd1 and the previously described Por1–Om14 complex in the mitochondrial outer membrane (Lauffer et al., 2012; Sakaue et al., 2019). This new contact site exists independently of contact sites formed by MICOS.

### Overexpression of *CQD1* leads to altered mitochondrial architecture and morphology

**Fig. 7.**
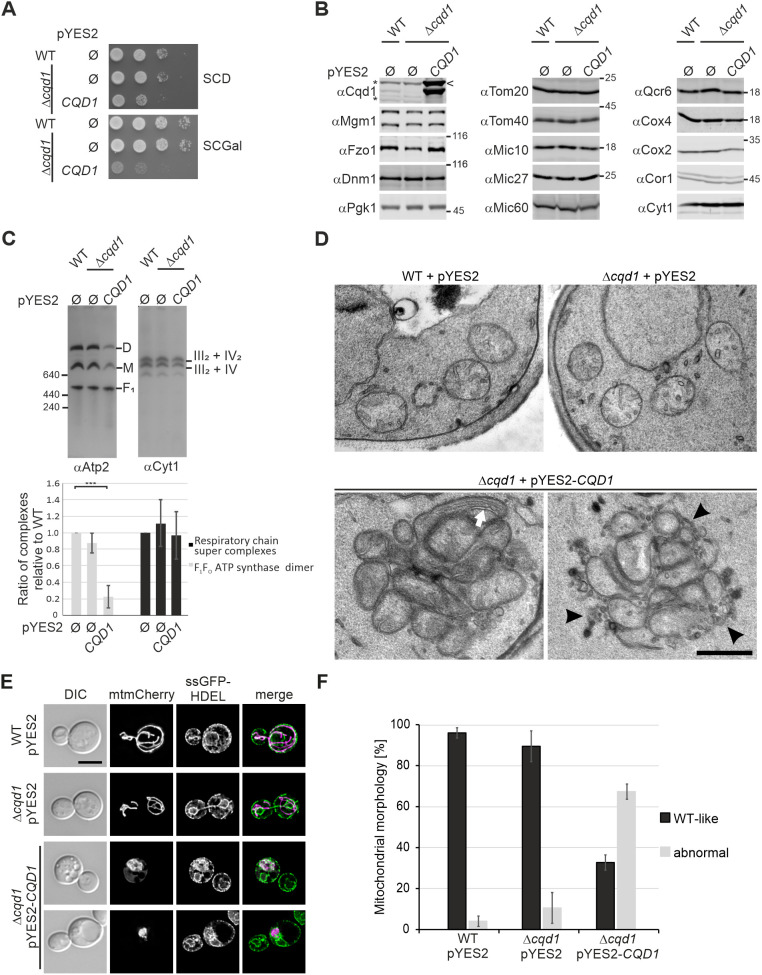
**Overexpression of *CQD1* leads to dramatic changes in architecture and morphology of mitochondria.** (A) Overexpression of *CQD1* is toxic. Wild-type and Δ*cqd1* cells carrying an empty vector (pYES2 Ø) and Δ*cqd1* cells expressing *CQD1* from a high-copy plasmid with a galactose-inducible promoter (pYES2-*CQD1*) were grown in SCD and shifted to SCGal prior to analysis. (B) Deletion or overexpression of *CQD1* does not affect steady-state levels of mitochondrial proteins. Whole-cell extracts of wild-type and yeast cells in which *CQD1* was deleted or overexpressed were analyzed by immunoblotting. Asterisks indicate cross reactions of the anti-Cqd1 antibody. The arrowhead indicates the potential precursor form of Cqd1. (C) Overexpression of Cqd1 results in strongly reduced levels of assembled F_1_F_O_ ATP synthase. Isolated mitochondria of cells grown in SCGal were lysed in buffer containing digitonin (3% w/v) and cleared lysates were subjected to BN-PAGE. The assembly of the F_1_F_O_ ATP synthase or the respiratory chain super complexes was analyzed by immunoblotting using antibodies against Atp2 or Cyt1. D, dimer of the F_1_F_O_ ATP synthase; M, monomer of the F_1_F_O_ ATP synthase; *F*_1_, *F*_1_ subcomplex of the F_1_F_O_ ATP synthase. Upper panel, immunoblot for one representative experiment. Lower panel, quantification of signal intensities of the F_1_F_O_ ATP synthase dimers and the respiratory chain super complexes [complex III dimer–complex IV monomer (III_2_–IV) and complex III dimer–complex IV dimer (III_2_-IV_2_)] present in the indicated strains of three biological replicates as determined by Image J software. Error bars indicate s.d. ***P≤0.001 (one-way ANOVA with subsequent Tukey's multiple comparison test). (D) Overexpression of *CQD1* results in highly altered mitochondrial architecture. Cells were grown overnight in SCGal, subjected to chemical fixation with glutaraldehyde and osmium tetroxide, embedded in Epon, and ultrathin sections were analyzed by transmission electron microscopy. The white arrow highlights elongated inner membrane structures; black arrowheads highlight membranes presumably representing cross-sections of ER tubules. Scale bar: 500 nm. Additional electron micrographs are shown in Fig. S2. (E) Overexpression of *CQD1* leads to the formation of mitochondria–ER clusters. Strains expressing mitochondria-targeted mCherry (mtmCherry) and ER-targeted GFP (ssGFP-HDEL) were grown to logarithmic growth phase in SCGal, fixed with formaldehyde, and examined by deconvolution fluorescence microscopy. Shown are maximum intensity projections of *z* stacks of entire cells (mitochondria) or of the center of the cells (ER, four consecutive *z* sections). DIC, differential interference contrast*.* Scale bar: 5 μm. (F) Strains expressing mitochondria-targeted GFP were analyzed as in E and mitochondrial morphology was quantified. Columns represent mean±s.d. values from two independent experiments with three biological replicates per strain and at least 150 cells per replicate. Representative images are shown in Fig. S3. Images in B and D are representative of at least three repeats.

Cells overexpressing *CQD1* from the strong *GAL* promoter showed a dramatically reduced growth rate (Fig. 7A). Western blot analysis using a Cqd1 specific antibody revealed the appearance of a slower migrating band, probably representing the accumulation of some Cqd1 precursor protein, upon overexpression (Fig. 7B, arrowhead). The levels of several other mitochondrial proteins involved in mitochondrial dynamics, protein import, mitochondrial architecture or respiration were not changed compared to those seen in the wild-type and the Δ*cqd1* deletion mutant (Fig. 7B). However, when we analyzed the presence of different mitochondrial protein complexes, we found that monomeric and dimeric F_1_F_O_ ATP synthase complexes were strongly reduced. In contrast, respiratory chain super complexes were not significantly changed (Fig. 7C).

The F_1_F_O_ ATP synthase is essential for the establishment of mitochondrial inner membrane architecture (Klecker and Westermann, 2021). Absence of assembled, dimeric F_1_F_O_ ATP synthase results in the formation of septa that cross the matrix completely, or onion-like structures (Davies et al., 2012; Habersetzer et al., 2013; Harner et al., 2016; Paumard et al., 2002; Rabl et al., 2009). Therefore, we asked whether the lack of assembled F_1_F_O_ ATP synthase complexes in the *CQD1* overexpression strain results in altered mitochondrial architecture. Electron micrographs of Δ*cqd1* deletion revealed they had a wild-type appearance, whereas overexpression of *CQD1* led to massively distorted mitochondrial membrane architecture in many cells (Fig. 7D; Fig. S2). Mitochondria were often aggregated into huge clusters, and we frequently observed loss of normal cristae, in many cases accompanied by the formation of elongated inner membrane structures (Fig. 7D, arrow; Fig. S2). Thus, aberrant mitochondrial architecture upon overexpression of *CQD1* complements the loss of assembled F_1_F_O_ ATP synthase.

Furthermore, in several cases we noticed membranous structures within these clusters that might represent ER membranes (Fig. 7D, arrowheads; Fig. S2). To test this, we simultaneously analyzed the morphology of mitochondria and the ER by fluorescence microscopy in wild-type and the *CQD1* deletion and overexpression strains. When *CQD1* was overexpressed, mitochondria in most cells did not appear as the characteristic tubular network that is spread all over the cell, but instead formed swollen clusters (Fig. 7E,F; Fig. S3). Remarkably, these clusters indeed contained a large proportion of ER membranes (Fig. 7E).

In summary, overexpression of *CQD1* causes severe phenotypes on growth, assembly of the F_1_F_O_ ATP synthase, and mitochondrial architecture and morphology. Interestingly, the effect of overexpression of the inner membrane protein Cqd1 is apparently not only restricted to the inner membrane but also affects inter-organellar contacts.

### Cqd1 and Cqd2 accumulate in the outer membrane upon overexpression

**Fig. 8.**
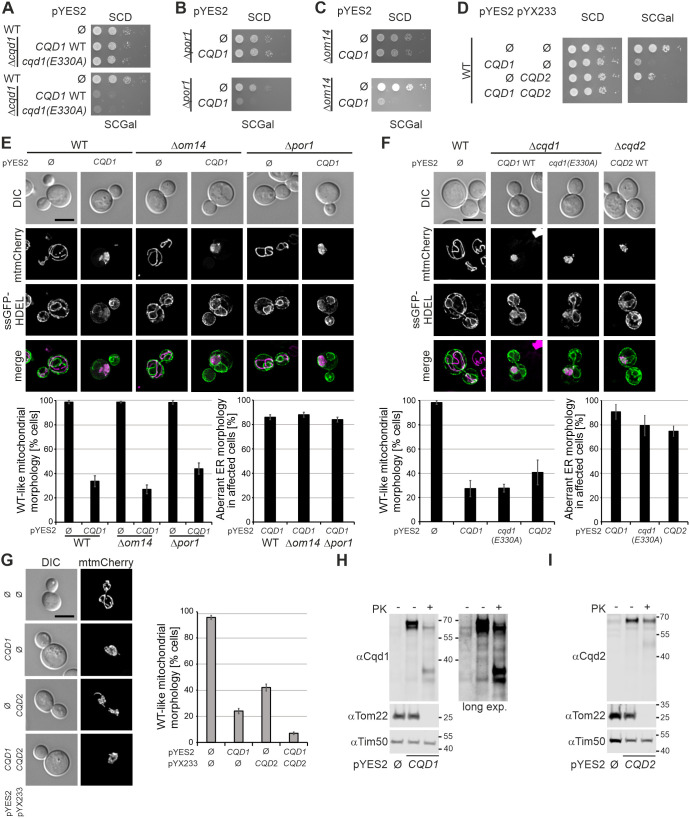
**The *CQD1* and *CQD2* overexpression phenotypes might be a result of altered topology.** (A) Overexpression of the nonfunctional *cqd1(E330A)* allele is toxic. Wild-type cells carrying an empty pYES2 plasmid (Ø) and Δ*cqd1* cells carrying pYES2 plasmids to overexpress *CQD1 WT* or *cqd1(E330A)* were grown in SCD and shifted to SCGal prior to analysis. Growth was analyzed by drop dilution assay on SCD and SCGal. (B,C) Om14 and Por1 are not required for the growth defect caused by *CQD1* overexpression. Cells of the indicated strains expressing *CQD1* at wild-type level or overexpressing *CQD1* were analyzed as in A. (B) Analysis of the *CQD1* overexpression phenotype in the Δ*por1* background. (C) Analysis of the *CQD1* overexpression phenotype in the Δ*om14* background. (D) Simultaneous overexpression of *CQD1* and *CQD2* is almost lethal. Growth of wild-type cells carrying the indicated pYES2 and pYX233 plasmids to overexpress *CQD1* and/or *CQD2* was analyzed as in A. (E) Overexpression of *CQD1* results in the formation of mitochondria–ER clusters in absence of Om14 and Por1. The indicated strains expressing mtmCherry and ssGFP-HDEL were grown to logarithmic growth phase in SCGal, fixed with formaldehyde, and examined by deconvolution fluorescence microscopy. Upper panel, maximum intensity projections of *z* stacks of entire cells (mitochondria) or of the center of the cells (ER, five consecutive *z* sections). DIC, differential interference contrast*.* Scale bar: 5 μm. Lower panel, quantitative evaluation of mitochondrial and ER morphologies. Columns represent mean±s.d. values from three independent experiments. In each experiment, mitochondrial morphology of at least 100 cells per strain was quantified. For the overexpression strains, also ER morphology of at least 50 cells with altered mitochondrial morphology was analyzed. (F) Overexpression of *cqd1(E330A)* and *CQD2* also leads to the generation of mitochondria–ER clusters. The indicated strains were analyzed as in E. (G) Simultaneous overexpression of *CQD1* and *CQD2* exacerbates the mitochondrial morphology defect phenotype. Mitochondrial morphology of the indicated strains expressing mtmCherry was analyzed as in E. (H) Overexpression of *CQD1* leads to an altered topology. Intact mitochondria from cells expressing *CQD1* at wild-type level or overexpressing *CQD1* were left untreated or were treated with proteinase K (PK). The indicated fractions were analyzed by SDS-PAGE and immunoblotting. Two different exposures of the anti-Cqd1 decoration are shown to better visualize Cqd1 at wild-type level. (I) Overexpression of *CQD2* leads to an altered topology as well. Intact mitochondria from cells expressing *CQD2* at wild-type level or overexpressing *CQD2* were treated as in (H). Images in A–D, H and I are representative of at least three repeats.

Next, we asked whether an intact protein kinase-like domain of Cqd1 or contacts with Por1 or Om14 are required for the overexpression phenotype. Surprisingly, overexpression of *cqd1(E330A)* was also toxic to the cells (Fig. 8A) and toxicity of Cqd1 did not require the presence of Por1 (Fig. 8B) or Om14 (Fig. 8C). Next, we tested whether *CQD1* and *CQD2* act antagonistically upon overexpression. Interestingly, we found that the overexpression of *CQD2* also reduces cell growth. Instead of having a compensatory effect, the simultaneous overexpression of both proteins exacerbated the growth defect, and cells were almost inviable (Fig. 8D). Consistent with this, overexpression of *CQD1* in the presence or absence of Por1 and Om14 (Fig. 8E) as well as overexpression of *cqd1(E330A)* or *CQD2* (Fig. 8F) led to very similar mitochondrial morphology defects. These were even more severe upon simultaneous overexpression of *CQD1* and *CQD2* (Fig. 8G). Moreover, ER morphology was aberrant in cells overexpressing *CQD1*, *cqd1(E330A)* or *CQD2*. These observations suggest that growth defects, aberrant mitochondrial morphology and increased ER contacts caused by excess Cqd1 are not dependent on its interaction with Por1 or Om14 and are not antagonized by Cqd2.

It was surprising that *CQD1* and *CQD2* overexpression induced aberrant ER morphology, as Cqd1 (Fig. 1B,C) and Cqd2 (Tan et al., 2013) normally are located in the mitochondrial inner membrane where they cannot establish direct contacts with the ER. Overexpression of *CQD1* resulted in the accumulation of a slower migrating band that might represent unprocessed precursor protein (Fig. 7B). This raised the possibility that altered topology is responsible for the overexpression phenotypes. Protease accessibility assays revealed that the slower migrating band appearing upon *CQD1* overexpression is present in the outer membrane (Fig. 8H). Overexpression of *CQD2* did not reveal a slower migrating band. However, a significant amount of Cqd2 was degradable by PK in intact mitochondria, whereas the inner membrane marker Tim50 remained stable (Fig. 8I), suggesting that also Cqd2 is present in the mitochondrial outer membrane upon overexpression.

In summary, it appears that both Cqd1 and Cqd2 accumulate in the outer membrane upon overexpression where they adopt a novel function that leads to clustering of mitochondria with the ER and severe growth defects. The establishment of inter-organellar contacts with the ER is consistent with the normal function of Cqd1 as a membrane contact site-forming protein in the inner membrane. This behavior explains very well the previously described suppressor function of *CQD2* overexpression in ERMES mutants (Tan et al., 2013). It is currently unclear whether a minor amount of Cqd1 or Cqd2 is also active in the mitochondrial outer membrane in wild-type cells under normal or stress conditions.

## DISCUSSION

In the present study, we characterized the highly conserved protein kinase-like domain containing protein Cqd1. All members of the UbiB family present in yeast, Cqd1, Cqd2 and Coq8, participate in coenzyme Q metabolism (Do et al., 2001; Johnson et al., 2005; Kemmerer et al., 2021; Reidenbach et al., 2018; Stefely et al., 2015; Subramanian et al., 2019; Xie et al., 2012). In contrast to the Δ*coq8* mutant, deletion of *CQD1* does not result in reduced production but affects distribution of coenzyme Q within the cellular membrane system (Kemmerer et al., 2021). The molecular role of Cqd1 in this process is unknown. However, membrane contacts would be ideally suited to facilitate the transition of these hydrophobic molecules (Kemmerer et al., 2021). The key finding of our study, the contact site formed by Cqd1, Por1 and Om14, is in good agreement with this hypothesis. Based on our results indicating that Cqd1 is also involved in phospholipid homeostasis, it is tempting to speculate that it might contribute to the import of phosphatidic acid, and possibly other lipids, into the inner membrane in addition to its role in coenzyme Q distribution. Interestingly, Cqd1 and Por1 share a number of common genetic interactors (Saccharomyces Genome Database, SGD; Cherry et al., 2012). These include genes encoding the ERMES subunits Gem1 and Mmm1 (Kornmann et al., 2009, 2011; Stroud et al., 2011), as well as Mdm35 and Psd1. In line with our hypothesis, all these proteins are involved in phospholipid homeostasis (Clancey et al., 1993; Kannan et al., 2015; Kojima et al., 2019; Miyata et al., 2016; Potting et al., 2010; Trotter et al., 1993; Watanabe et al., 2015; Yu et al., 2015).

We consider it unlikely that Cqd1 itself is a lipid transporter, as the absence of Cqd1 leads to increased export of coenzyme Q rather than block of transport (Kemmerer et al., 2021). However, it is conceivable that Cqd1 has an accessory or regulatory role and interacts with additional, as yet unknown, interaction partners in the inner membrane. This idea would be in line with models of other lipid transport systems, like the bacterial maintenance of lipid asymmetry (Mla) pathway first identified in *E. coli* (Malinverni and Silhavy, 2009). Here, the MlaFEDB complex is involved in the ATP-dependent transport of lipids between the bacterial inner and outer membranes (Malinverni and Silhavy, 2009; Thong et al., 2016). This complex is composed of the canonical proteins MlaE and MlaF and the auxiliary proteins MlaB and the hexameric MlaD in the inner membrane, and cooperates with MlaC, a soluble protein in the periplasm, and the outer membrane protein MlaA (Ekiert et al., 2017; Malinverni and Silhavy, 2009; Thong et al., 2016). Intriguingly, MlaA interacts with the porins OmpC and OmpF (Chong et al., 2015) and it was proposed that it works in both directions between the bacterial membranes (Ekiert et al., 2017; Malinverni and Silhavy, 2009). The Mla pathway represents a transport system based on transient protein interactions, in contrast to the here discovered permanent contact. However, it shares two striking features with Cqd1 – an interaction with porin across two membranes and the regulation of bi-directional lipid transport.

In addition to the observed changes in phospholipid composition upon deletion of *CQD1*, overexpression of *CQD1* results in altered mitochondrial architecture. Consistent with previous reports (Tan et al., 2013), we find that overexpression of *CQD2* leads to a similar phenotype. These changes are characterized by the formation of huge mitochondrial clusters. We observed that these mitochondrial clusters intimately interact with ER membranes, which supports a role of Cqd1 and Cqd2 in the establishment of membrane contact sites and might explain the previous finding (Tan et al., 2013) that the overexpression of *CQD2* can rescue the deletion of ERMES subunits. It remains a challenge for the future to explore whether Cqd1 and Cqd2 are, at least to a small extent, dually located in the mitochondrial inner and outer membranes where they might contribute to the passage of phosphatidic acid, coenzyme Q or other lipids between these organelles.

## MATERIALS AND METHODS

### Yeast strains and cell growth

*S. cerevisiae* YPH499 was used as wild-type (WT). Chromosomal manipulations (knockouts, C-terminal 3xHA and 3xMyc tagging) were performed according to established procedures (Knop et al., 1999; Longtine et al., 1998). For the generation of deletion strains, the entire coding regions of the corresponding genes were replaced by the indicated marker cassettes. Double mutant strains were also generated by homologous recombination. The genotypes of the strains used in this study are listed in Table S3. All strains used in this study can be requested from the corresponding author.

For the generation of pRS316-*CQD1*, pRS316-*CQD1*-3xHA, pYES2-*CQD1*, pYES2*-CQD2* and pXY233*-CQD2* plasmids, the coding region of *CQD1* or *CQD2* was amplified by PCR. The intron present in the *CQD1* open reading frame was removed when cloning the initial pYES2 construct (Invitrogen). This construct served as a template DNA for cloning of pRS316-*CQD1*. The coding region of *CQD1*-3xHA was amplified from yeast genomic DNA of the *CQD1*-3xHA strain. *CQD1*-3xHA was cloned under control of its endogenous promoter and the *ADH1* terminator into the pRS316 vector (Sikorski and Hieter, 1989) by enzymatic assembly of overlapping DNA fragments (Gibson, 2011). The point mutations K275A, D288A, and E330A in *CQD1* were introduced into pRS316-*CQD1* by site directed mutagenesis. For the generation of the pYX142-ssGFP-HDEL plasmid the *Sac*I/*Eco*RI fragment of pYX122-ssGFP-HDEL (Böckler and Westermann, 2014) was ligated into the *Sac*I/*Eco*RI sites of pYX142-mtGFP (Westermann and Neupert, 2000). The primers used in this study are listed in Table S4. All plasmids used in this study are either deposited at Addgene (plasmid 45050) or can be requested from the corresponding author.

Yeast cells were grown as indicated on YP medium supplemented with 2% glucose (YPD) or 3% glycerol (YPG) or synthetic medium supplemented with 2% glucose (SCD), 3% galactose (SCGal) or 3% glycerol (SCG) (Izawa and Unger, 2017). Culture components were from Merck (Darmstadt, Germany), Thermo Fisher Scientific (Waltham, MA, USA) and VWR international (Radnor, PA, USA).

Growth of the different strains was analyzed with a drop dilution assay. Cells were grown and kept in logarithmic growth phase, washed with water, diluted in water to an optical density of 600 nm (OD_600_) of 0.5 and serial dilutions were prepared (1:10; 1:100; 1:1000). 5 μl of each dilution were spotted on agar plates with the indicated medium. All strains without plasmids were grown initially in YPD liquid medium for 16 h. For analysis of cell growth on YP medium, cells were harvested after growth in YPD. When growth on synthetic medium was analyzed, cells were shifted from YPD to SCD 30 h prior to analysis. Cell carrying pYES2 plasmids were grown initially in SCD liquid medium for 16 h and shifted to SCGal for 7 h before growth was analyzed.

### Whole-cell extracts

Whole-cell extracts were prepared as described previously (Kushnirov, 2000). In brief, cells were grown in the indicated media to exponential growth phase (OD_600_ 0.5–1.0) and an equivalent of cells corresponding to 2.5 OD_600_ was collected by centrifugation (20,000 ***g*** for 1 min). Harvested cells were resuspended in 200 μl of 0.1 M NaOH and incubated at room temperature for 5 min. Cell pellets were resuspended in 50 μl of SDS sample buffer and analyzed by SDS-PAGE and immunoblotting (Fig. S4).

### Isolation of crude mitochondria

Cells were grown at 30°C in SCD, SCG, SCGal or YPG as indicated. Mitochondria were isolated by differential centrifugation as described previously (Izawa and Unger, 2017) with slight modifications. In brief, cells were harvested and washed with distilled water. Cells were treated for 10 min with DTT (10 mM final concentration) followed by treatment with zymolyase (200 U per gram wet weight of cells) for 30 min at 30°C with gentle agitation. Sphaeroplasts were opened by repeated pipetting in isotonic lysis buffer [20 mM MOPS-KOH pH 7.2, 1 mM EDTA, 0.6 M sorbitol, 0.2% (w/v) BSA and 1 mM PMSF]. Mitochondria were harvested after a clarifying spin at 2000 ***g*** and 4°C for 5 min by centrifugation at 14,000 ***g*** and 4°C for 10 min and resuspended in SM buffer (0.6 M sorbitol and 20 mM MOPS, pH 7.4).

### Sucrose gradient purification of mitochondria

Crude mitochondria were isolated as described above except that the final mitochondrial pellet was resuspended in 15 ml of buffer A (0.6 M Sorbitol, 50 mM MES pH 6.0) and re-isolated by centrifugation for 10 min at 17,000 ***g*** and 4°C. Crude mitochondria were resuspended in 2 ml of buffer A and 10 mg were loaded on top of a sucrose step gradient (1.5 ml of 60%, 5 ml of 35%, 1.5 ml of 25%, and 1.5 ml of 15% sucrose in buffer A). Mitochondria were separated from other organelles by ultracentrifugation for 1 h at 134,000 ***g*** and 4°C using a swinging-bucket rotor. Purified mitochondria were harvested from the boundary between 60% and 35% sucrose layers, resuspended in 15 ml of SM buffer and re-isolated by centrifugation for 15 min at 14,000 ***g*** and 4°C. Mitochondria were resuspended in SM buffer to a final concentration of 10 mg/ml.

### Subfractionation of mitochondria

Vesicles consisting of pure mitochondrial outer membrane, mitochondrial inner membrane, and vesicles consisting of both membranes were generated and separated as described before (Harner, 2017) with slight modifications. 10 mg of freshly isolated crude mitochondria were resuspended in 1.6 ml of SM buffer. Mitochondria were swollen by addition of 16 ml swelling buffer (20 mM MOPS pH 7.4) and subsequent incubation for 30 min at 4°C under mild stirring. Sucrose concentration was adjusted to 0.55 M. Vesicles were generated by sonication (four times 30 s at 10% amplitude intermitted by 30 s breaks). After a clarifying spin for 20 min at 20,000 ***g*** and 4°C, vesicles were concentrated on the 2.5 M sucrose cushion by centrifugation at 118,000 ***g*** and 4°C for 100 min. Concentrated vesicles were harvested and the suspension was homogenized by dounce homogenization. Vesicles were separated by centrifugation in a swinging-bucket rotor for 12 h at 200,000 ***g*** and 4°C through a sucrose-step gradient (0.8 M, 0.96 M, 1.02 M, 1.13 M, 1.25 M sucrose in 20 mM MOPS-KOH pH 7.4 and 0.5 mM EDTA). The gradient was harvested, and proteins were subjected twice to trichloroacetic acid precipitation (TCA; final concentration of 14.4%).

### Alkaline extraction

100 μg of isolated mitochondria were diluted in SM buffer to a concentration of 1 mg/ml. An equal volume of 200 mM sodium carbonate was added followed by an incubation on ice for 30 min. Membrane and soluble proteins were separated by centrifugation for 30 min at 91,000 ***g*** and 4°C. Proteins present in the supernatant were subjected to TCA precipitation. The membrane protein pellet and the precipitated soluble proteins were resuspended in SDS sample buffer and analyzed by SDS-PAGE and immunoblotting (Fig. S4).

### Proteolytic susceptibility assay

100 μg of mitochondria were incubated in SM buffer (0.6 M sorbitol, 20 mM MOPS, pH 7.4), swelling buffer (20 mM MOPS, pH 7.4) or lysis buffer [1% (v/v) Triton X-100, 20 mM MOPS, pH 7.4] for 20 min on ice. Proteinase K was added (final concentration of 0.2 mg/ml) as indicated, and samples were incubated on ice for 15 min. Proteolysis was stopped by addition of PMSF to a final concentration of 4 mM. Samples were centrifuged at 17,000 ***g*** and 4°C for 20 min. Pellets were resuspended in SM buffer and subjected to TCA precipitation. Samples were analyzed by SDS-PAGE and immunoblotting (Fig. S4).

### Blue-native gel electrophoresis

150 μg (MICOS complex) or 50 µg (all other mitochondrial protein complexes) of isolated mitochondria were resuspended in 15 μl (MICOS complex) or 50 μl (all other mitochondrial protein complexes) of Blue-native (BN) buffer (50 mM NaCl, 50 mM imidazole-HCl, 2 mM 6-aminohexanoic acid, 1 mM EDTA, 1 mM PMSF, pH 7.0) (Wittig et al., 2006) supplemented with 3% digitonin. After a clarifying spin for 15 min at 14,000 ***g*** and 4°C, 2 μl (MICOS complex) or 7 μl (all other mitochondrial protein complexes) G-250 Sample Additive (Invitrogen, Waltham, Massachusetts, USA) containing 40% glycerol were added. Protein complexes were separated on 3–12% native PAGE Novex Bis-Tris gels (Invitrogen). Electrophoresis was performed for 30 min at 150 V followed by 2 h at 250 V. Proteins were transferred to PVDF membrane by wet blotting for 2 h at 30 mV.

### Chemical crosslinking

Isolated mitochondria were diluted in 100 μl of SI buffer (50 mM HEPES-KOH, 0.6 M sorbitol, 75 mM KCl, 10 mM Mg(Ac)_2_, 2 mM KH_2_PO_4_, 2.5 mM EDTA and 2.5 mM MnCl_2_, pH 7.2) to a concentration of 1 mg/ml. Disuccinimidyl glutarate (DSG; Thermo Fisher Scientific) and m-maleimidobenzoyl-N-hydroxysuccinimide ester (MBS; Thermo Fisher Scientific) were added from freshly prepared stock solutions to the final concentration of 200 or 400 μM and incubated on ice for 30 min. The reaction was stopped by the addition of glycine to a final concentration of 100 mM followed by incubation on ice for 10 min. Mitochondria were re-isolated by centrifugation at 17,000 ***g*** and 4°C for 10 min. The pellets were resuspended in SDS sample buffer and analyzed by SDS-PAGE and immunoblotting (Fig. S4).

### Immunoprecipitation assay

3 mg mitochondria (except for Cqd1-3×HA, 1 mg of mitochondria) were lysed in IP buffer (50 mM Tris-HCl pH 7.4, 50 mM NaCl) containing 1% (w/v) digitonin and 1 mM PMSF. After a clarifying spin for 10 min at 12,000 ***g*** and 4°C, lysates were incubated with anti-HA agarose beads (20 μl of beads per 1 mg of proteins) (Merck) for 2 h. Anti-HA agarose beads were washed three times with 500 µl IP buffer containing 0.1% (w/v) digitonin and 1 mM PMSF before and after incubation with lysates. The indicated amount of total lysate and unbound material was taken, subjected to TCA precipitation and resuspended in SDS sample buffer. Bound proteins were eluted with SDS sample buffer at 95°C for 10 min. Fractions were analyzed by SDS-PAGE followed by immunoblotting (Fig. S4).

### Antibodies against Cqd1 and Cqd2

Antibodies against yeast Cqd1 and Cqd2 were generated in rabbits by injection of recombinant proteins comprising amino acid residues 143–571 (Cqd1) or 53–415 (Cqd2).

For affinity purification the respective antigens were coupled to CNBr-activated Sepharose 4B beads (5 mg protein/ml bead volume; Merck) in 0.1 M NaHCO_3_, 0.5 M NaCl, pH 8.3. 6 ml Cqd1 or Cqd2 antiserum diluted in 24 ml 10 mM Tris-HCl pH 7.5 containing 1 mM PMSF and 1 mM EDTA were applied to the respective beads and washed with 10 bead volumes 10 mM Tris-HCl pH 7.5 followed by 10 bead volumes 10 mM Tris-HCl pH 7.5, 0.5 M NaCl. Antibodies were eluted using 10 bead volumes of 100 mM sodium citrate, pH 4.0 followed by 10 bead volumes of 100 mM glycine, pH 2.5. Elution was collected in 1 ml fractions and immediately neutralized by addition of 200 µl 1 M Tris-HCl pH 8.8. The specificity of the antibodies was evaluated by immunoblotting (Figs 2D, 7B, 8H and I). All additional antibodies used in this study are listed in Table S5.

### Fluorescence microscopy

For visualizing mitochondria, the mitochondrial presequence of *Neurospora crassa* subunit 9 of the F_1_F_O_ ATP synthase was fused to mKate2. The nucleotide sequence was inserted into the HO locus of the yeast genome and expressed constitutively under the control of the *PGK1* promoter. Yeast cells were grown overnight in YPD at 30°C. The next day, cells were diluted in SCD medium and were kept in logarithmic phase for 24 h. Cells corresponding to an OD_600_ of 1 were harvested by centrifugation at 2500 ***g*** for 3 min. Cells were vortexed for 1 min and washed with 1 ml of sterile 1× PBS. Cell pellets were resuspended in 200 μl 1× PBS and immobilized on μ-slides (Ibidi, Gräfelfing, Germany) coated with 1 mg/ml of concanavalin A (Merck). After immobilizing, cells were covered with 400 μl SCD medium. Microscopy was performed at 30°C on a Nikon Ti2-Eclipse microscope (Nikon, Tokyo, Japan) equipped with a CFI Apochromat TIRF 100×/1.49 NA oil objective and a TwinCam LS dual camera splitter attached to two Photometrics Prime 95B 25 mm cameras (Teledyne Photometrics, Tucson, USA).

For visualization of mitochondria or mitochondria and the ER upon *CQD1* and/or *CQD2* overexpression, strains carried either pYX142-mtGFP (Westermann and Neupert, 2000), pYX232-mtmCherry or pYX122-mtmCherry (Dirk Scholz, University of Bayreuth) and pYX142-ssGFP-HDEL or pYX122-ssGFP-HDEL (Böckler and Westermann, 2014), respectively. Cells were grown overnight in galactose-containing synthetic complete medium supplemented with 0.1% glucose, shifted to galactose-containing synthetic complete medium without glucose, incubated until logarithmic growth phase and fixed with 3.7% formaldehyde. Microscopy was performed using a Leica DMi8 fluorescence microscope (Leica Microsystems GmbH, Wetzlar, Germany) equipped with a HC PL APO 100×/1.40 oil objective, a Lumencor SPECTRA X light source and fluorescence filter sets (TXR Cube excitation 540–580 nm; emission 592–668 nm and FITC Cube excitation 460–500 nm; emission 512–542 nm). Images were taken with a Leica DFC9000 GT VSC-07400 sCMOS camera. For microscope settings, image generation, and processing (cropping, maximum intensity projection), the Leica LAS X software (version 3.6.0.20104, Leica Microsystems GmbH, Wetzlar, Germany) was used. For deconvolution of *z*-stacks the Deconvolution Software Huygens Essential (version 18.10, Scientific Volume Imaging, Hilversum, The Netherlands) was used. For adjustment of brightness and contrast and the overlay of different channels, Adobe Photoshop CS6 (Adobe Systems) was used. For simultaneous analysis of the morphology of mitochondria and the ER, *z* stacks were recorded with a *z*-step size of 213 nm. In the case of mitochondria, maximum intensity projections of *z* stacks of entire cells are shown, whereas for the ER maximum intensity projections of either four or five consecutive *z* sections of the center of the cells are shown.

### Electron microscopy

For electron microscopy, cells were first grown in synthetic complete medium containing glucose as the carbon source and then shifted to synthetic complete medium containing galactose as carbon source, in which they were grown overnight. Chemical fixation of yeast cells with glutaraldehyde and osmium tetroxide, dehydration, Epon embedding and subsequent steps of sample preparation for electron microscopy were performed as described previously (Unger et al., 2017). Electron micrographs were taken with a JEOL JEM-1400 Plus transmission electron microscope operated at 80 kV, a JEOL Ruby CCD camera (3296×2472 pixels), and the TEM Center software Ver.1.7.12.1984 or Ver.1.7.19.2439 (JEOL, Tokyo, Japan).

### Lipid analysis

Lipidomics analyses were performed as described in Papagiannidis et al. (2021). Aliquots corresponding to 1500–2000 pmol total lipid were subjected to acidic Bligh and Dyer extractions performed in the presence of internal lipid standards from a master mix containing 40 pmol d_7_-phosphatidylcholine (PC) mix (15:0/18:1-d7, Avanti Polar Lipids), 25 pmol phosphatidylinositol (PI; 17:0/20:4, Avanti Polar Lipids), 25 pmol phosphatidylethanolamine (PE) and 15 pmol phosphatidylserine (PS; 14:1/14:1, 20:1/20:1, 22:1/22:1, semi-synthesized as described in Özbalci et al., 2013), 20 pmol PA (PA 17:0/20:4, Avanti Polar Lipids), 5 pmol phosphatidylglycerol (PG; 14:1/14:1, 20:1/20:1, 22:1/22:1, semi-synthesized as described in Özbalci et al., 2013), 25 pmol cardiolipin (CL) 57:4 (14:1/14:1/14:1/15:1, Avanti Polar Lipids) and 25 pmol monolysocardiolipin (MLCL; 16:0/16:0/16:0, Echelon). Lipids recovered in the organic extraction phase were evaporated by a gentle stream of nitrogen. Prior to measurements, lipid extracts were dissolved in 10 mM ammonium acetate in methanol, diluted 1:10 and transferred into Eppendorf twin.tec 96-well plates. Mass spectrometric measurements were performed in positive ion mode on an AB SCIEX QTRAP 6500+ mass spectrometer equipped with chip-based (HD-D ESI Chip, Advion Biosciences) nano-electrospray infusion and ionization (Triversa Nanomate, Advion Biosciences) as described (Özbalci et al., 2013). The following precursor ion scanning (PREC) and neutral loss scanning (NL) modes were used for the measurement of the various lipid classes: +PREC 184 (PC), +NL141 (PE), +NL185 (PS), +NL277 (PI), +NL189 (PG) and, +NL115 (PA). Mass spectrometry settings were: Resolution: unit, low mass configuration; data accumulation: 400 MCA; curtain gas: 20; interface heater temperature: 60°C; CAD: medium. Data evaluation was performed using LipidView (Sciex) and ShinyLipids, a software developed in-house. For MS analysis of CL and MLCL, dried lipids were re-dissolved in 40% UPLC solvent B (90% 2-propanol, 10% acetonitrile, 0.1% formic acid and 10 mM NH_4_HCO_3_) and transferred to silanized glass inserts (Phenomenex) using Hamilton syringes. The glass inserts were placed in Eppendorf tubes and centrifuged in an Eppendorf centrifuge at 745 ***g*** for 3 min. Lipid samples were then subjected to UPLC-ESI-MS/MS analysis performed on an Ultimate® 3000 LC system (Dionex, Thermo Fisher Scientific) coupled to a QExactive Hybrid quadrupole-orbitrap instrument (Thermo Fisher Scientific). For LC separations, an ACQUITY UPLC CSH C18 1.7 µm, 1.0×150 mm column (Waters) was used. The column oven temperature was set to 55°C, the temperature of the autosampler was set to 20°C. The flow rate used was 100 µl/min. The solvent composition used was as follows: 60% acetonitrile, 40% H_2_O, 0.1% formic acid and 10 mM NH_4_HCO_3_ (solvent A), and 90% 2-propanol, 10% acetonitrile, 0.1% formic acid and 10 mM NH_4_HCO_2_ (solvent B). The starting solvent composition was 50% solvent B:50% solvent A. The conditions of the gradient were as follows: 7 min, 90% solvent B; 7–17 min, 90% solvent B; 17.1 min, 50% solvent B; and 17.1–25 min; 50% solvent B. The MS analyses were performed in the positive ion mode. CL and MLCL species were measured as [M+NH_4_]^+^ ions. The following ESI source parameters were used: sheath gas flow rate: 4 (a.u.), auxiliary gas flow rate: 0, sweep gas flow rate: 0, spray voltage: 1.5 kV, capillary temperature: 200°C, S-lens RF level: 50. Full-MS scans were recorded using the following parameters: resolution: 140,000 (at *m*/*z* 200) AGC-target: 1e6, maximum IT: 200 ms, scan range: *m*/*z* 500-2000. Data evaluation was performed using MassMap.

### Statistics

Statistics were calculated using the software GraphPad Prism (version 5; GraphPad, San Diego, CA, USA). Mass spectrometry analysis was performed in quadruplicate. Data are represented as mean±s.d. For statistical analysis, data was first tested for normal distribution using Shapiro–Wilk normality test. *P*-values were calculated using a Mann–Whitney test. Analysis of Mdj1 precursor accumulation and Mgm1 processing in the various yeast strains was performed in triplicate. Data are represented as mean±s.d. *P*-values were calculated using an unpaired two-tailed Student's *t*-test. Analysis of the steady state level of Cqd1 variants expressed from pRS316 under the control of the endogenous *CQD1* promotor as well as the level of assembled mitochondrial protein complexes were performed in triplicates. Data are represented as mean±s.d. *P*-values were calculated using one-way ANOVA test with subsequent Tukey's multiple comparison test. *P*<0.05 was considered statistically significant.

## Supplementary Material

10.1242/joces.260578_sup1 Supplementary information
