# Supplementary material for: The UbiB family member Cqd1 forms a novel membrane contact site in mitochondria
Source: J Cell Sci. 2023 May 18;136(10):jcs260578. doi: 10.1242/jcs.260578 (PMC10214851; doi:10.1242/jcs.260578)
Supplement: Supplementary information [file joces-136-260578-s1.pdf]

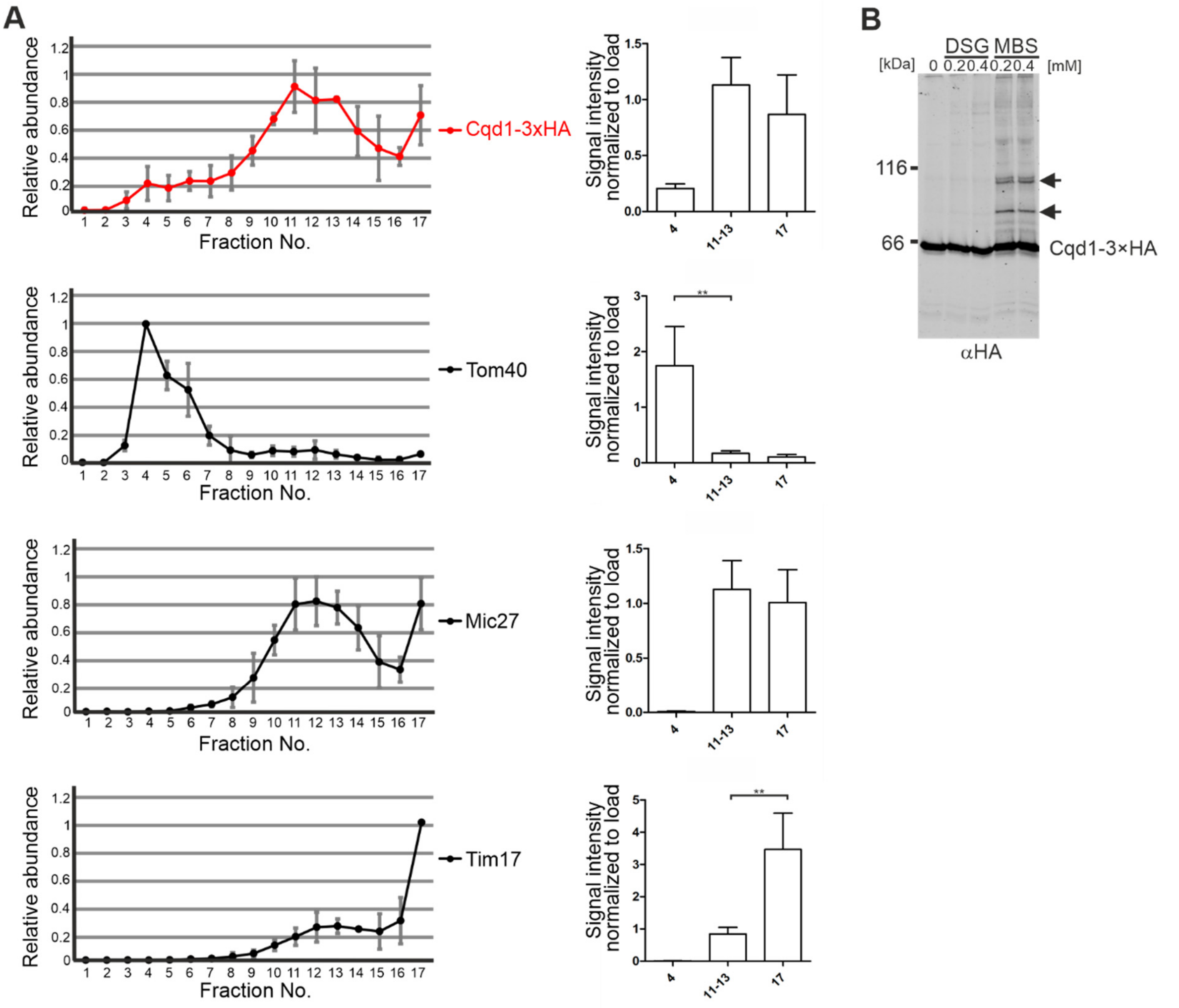

Fig. S1. Cqd1 is enriched in fractions containing contact site proteins.

(A) (Left) The average of relative protein abundance in three independent experiments described in Fig. 6A is shown. The graph shows mean values of the distribution of Cqd1-3xHA and the marker proteins for the outer membrane (Tom40), the inner membrane (Tim17) and contact sites (Mic27). Error bars indicate standard deviation. (Right) The quantification of the amount of the respective proteins present in low density fractions (No. 4), intermediate density fractions (No. 11-13) and high density fractions (No. 17) is shown as mean from three independent experiments. Quantification was done using Image Studio software. Error bars indicate standard deviation. Asterisks represent p-values obtained by unpaired Student's t test (**p≤0.01). **(B)** Cqd1 interacts with proteins other than itself. Isolated mitochondria of a Cqd1-3xHA expressing strain were exposed to DMSO or the chemical crosslinkers DSG and MBS at the indicated concentrations. Samples were analyzed by immunoblotting with an anti-HA antibody. Arrows indicate Cqd1-containing crosslinks.

Journal of Cell Science • Supplementary information

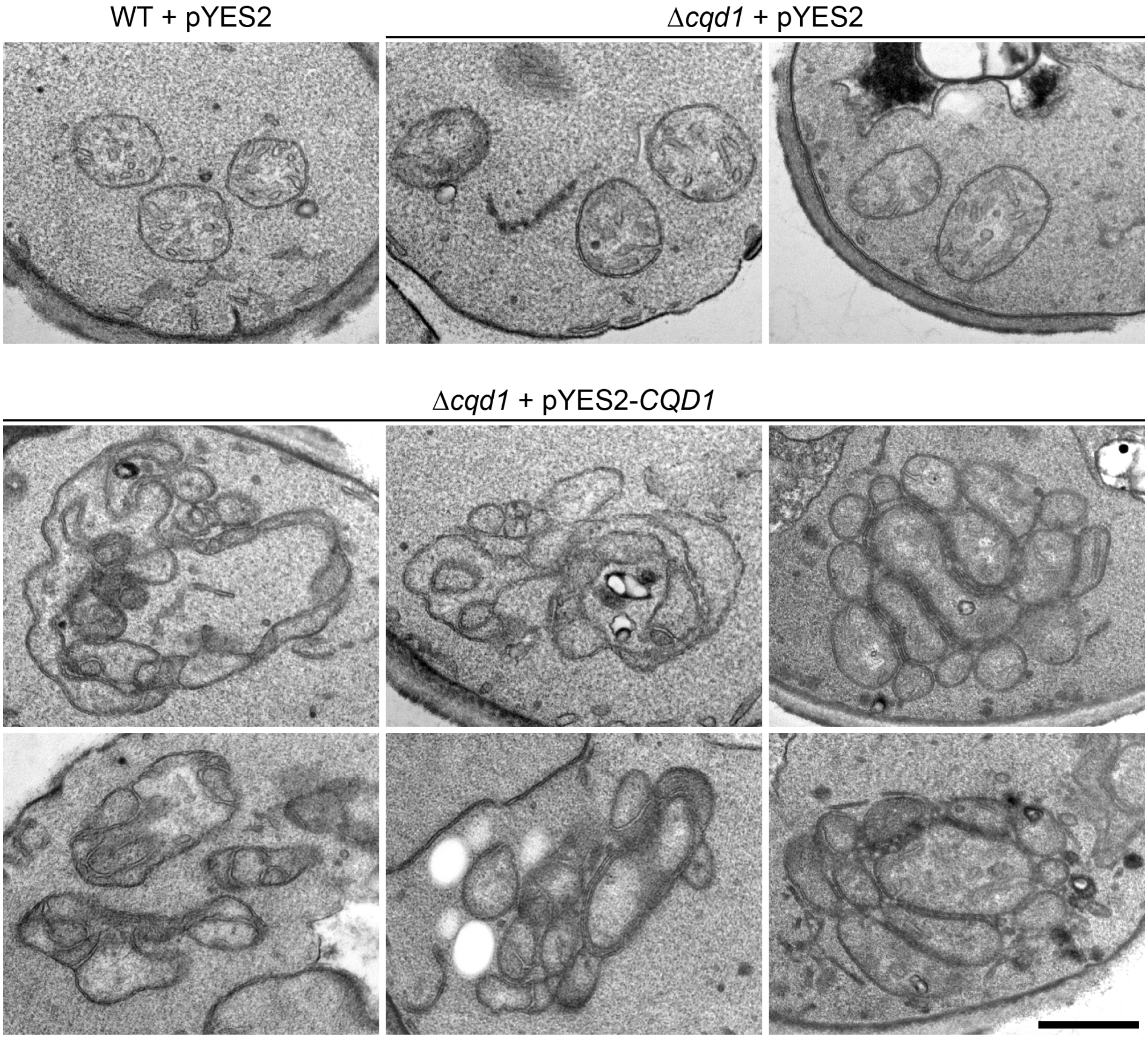

Fig. S2. *CQD1* overexpression is associated with altered mitochondrial architecture. WT and Δ*cqd1* cells carrying the indicated plasmids were grown and analyzed by electron microscopy as described in Fig. 7D. Scale bar, 500 nm.

Journal of Cell Science • Supplementary information

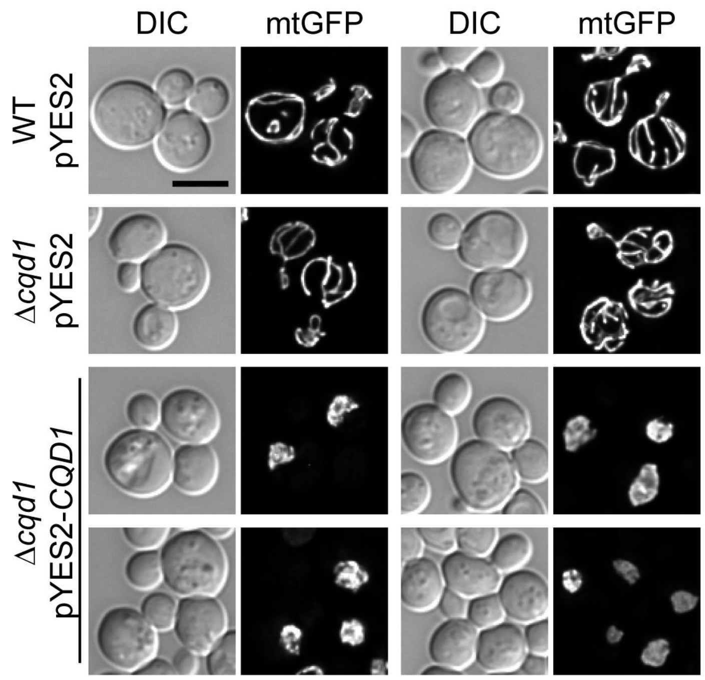

Fig. S3. Overexpression of *CQD1* causes changes in mitochondrial morphology.

WT and Δ*cqd1* cells carrying the indicated plasmids and expressing mitochondria-targeted GFP (mtGFP) were grown and analyzed as described in Fig. 7E. Shown are DIC (differential interference contrast) images and maximum intensity projections of deconvolved z stacks (mtGFP) from representative cells. Scale bar, 5 µm.

Fig. 1B

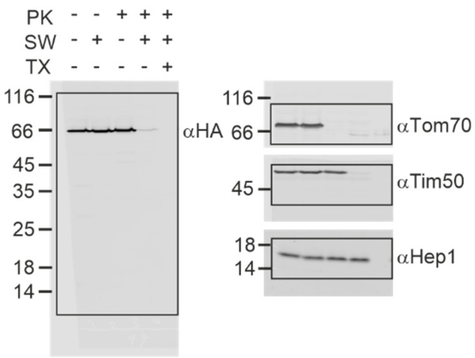

Fig. 1C

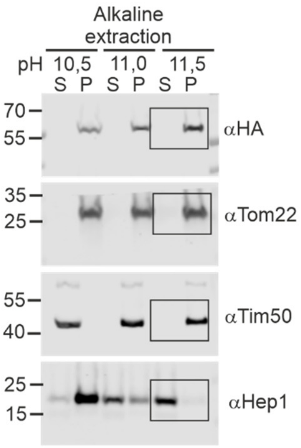

Fig. 2D

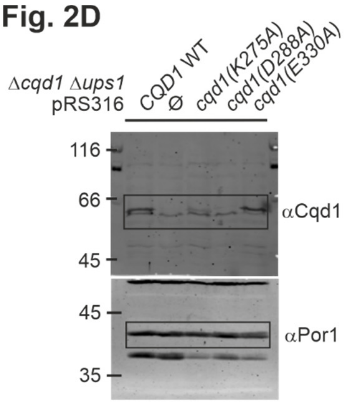

Fig. 4A

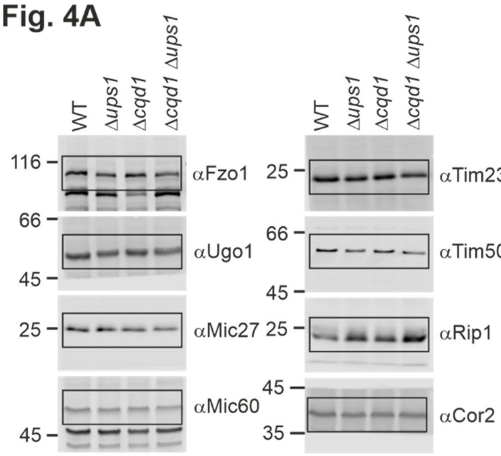

Fig. 4B

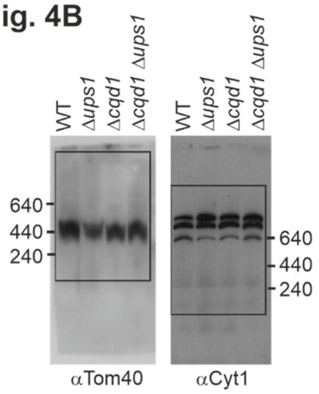

Fig. 4C

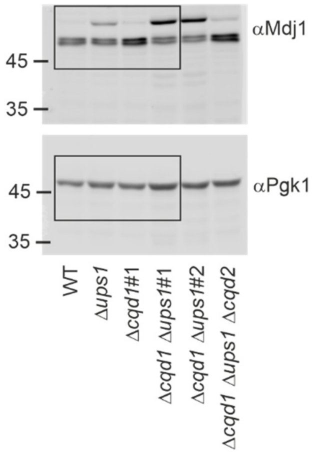

Fig. 4D

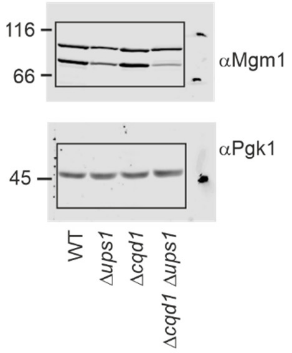

Fig. 5B

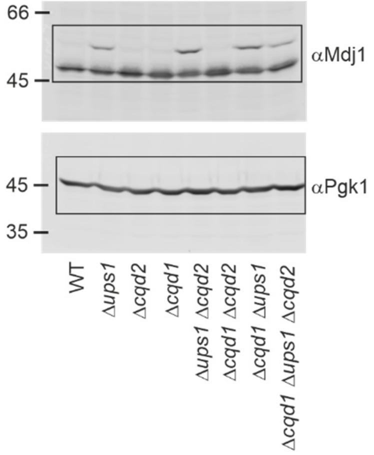

Fig. 5C

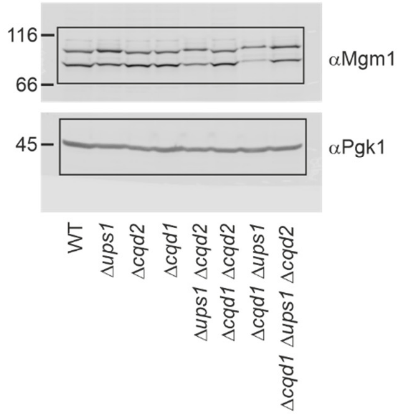

Journal of Cell Science • Supplementary information

Fig. 6A

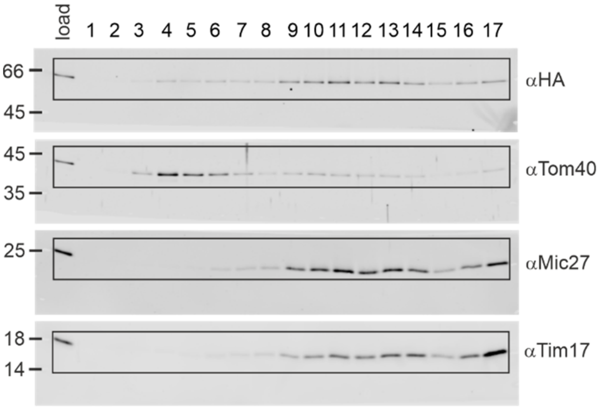

Fig. 6B

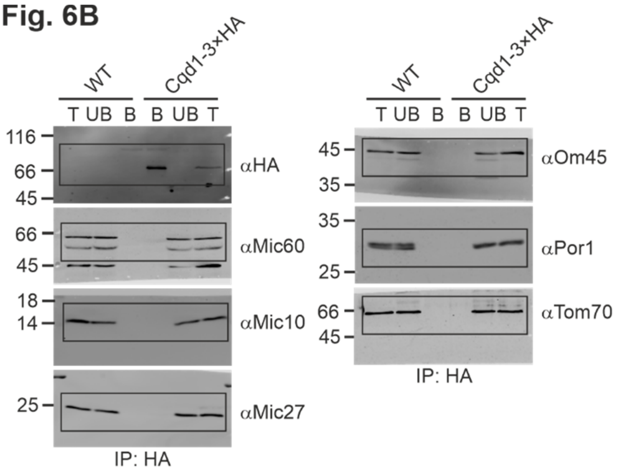

Fig. 6C

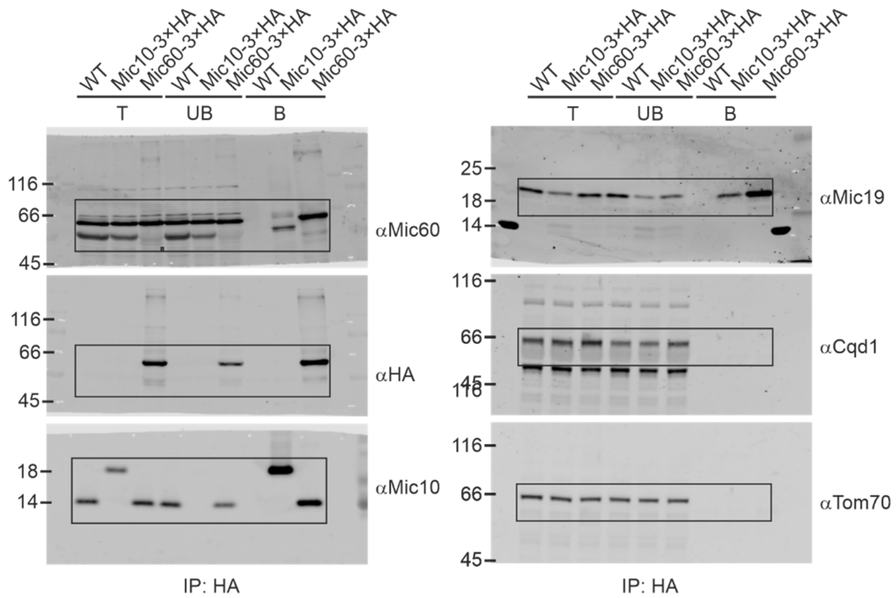

Fig. 6D

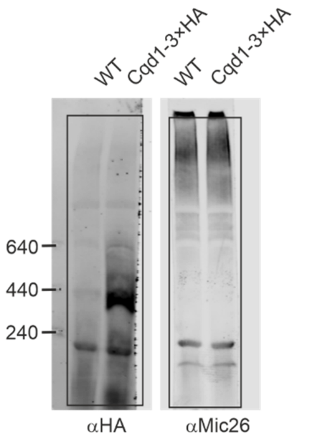

Journal of Cell Science • Supplementary information

Fig. 6E

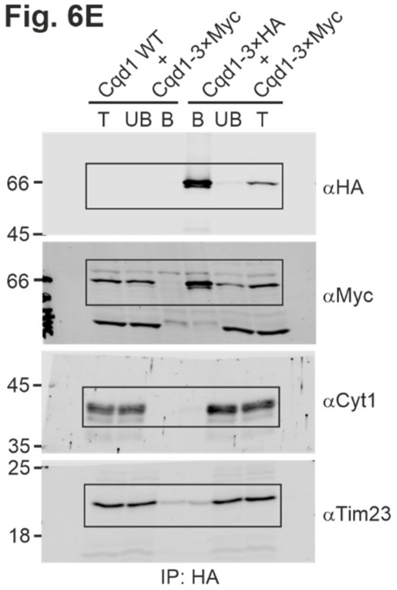

Fig. 6F

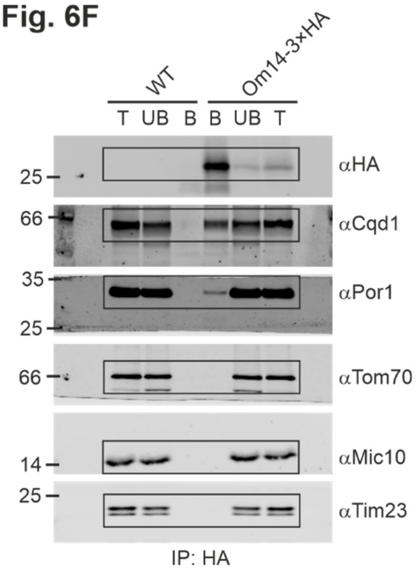

Fig. 6G

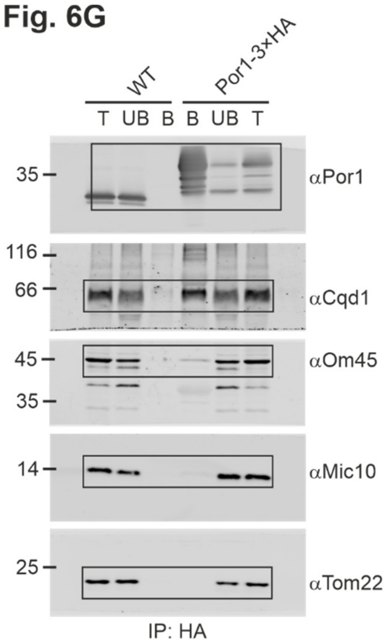

Fig. 6H

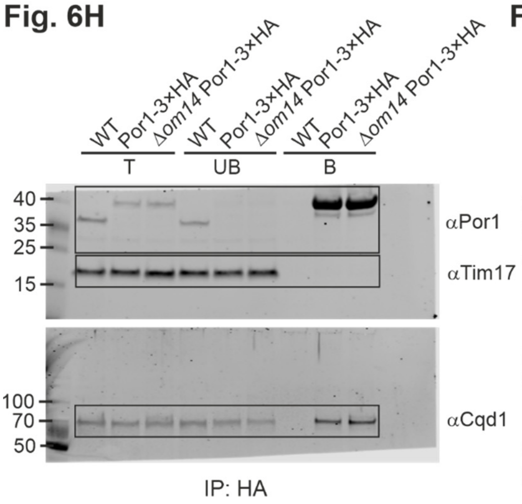

Fig. 6I

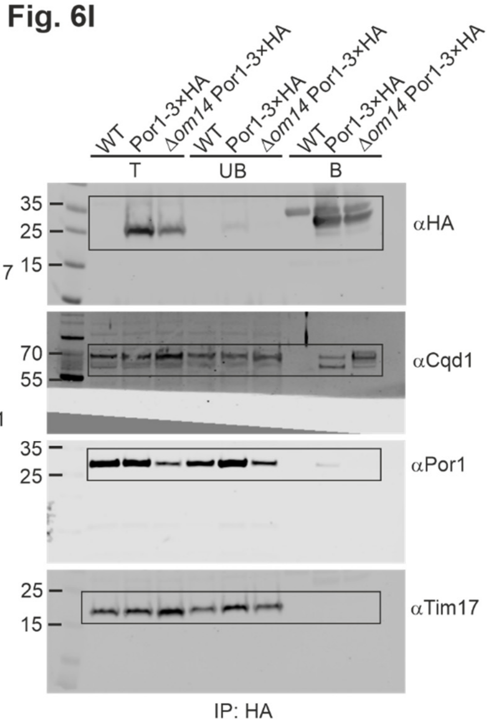

Journal of Cell Science • Supplementary information

Fig. 6J

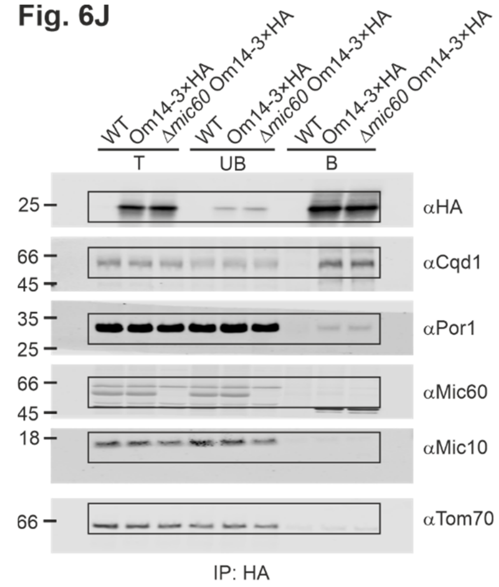

Fig. 7B

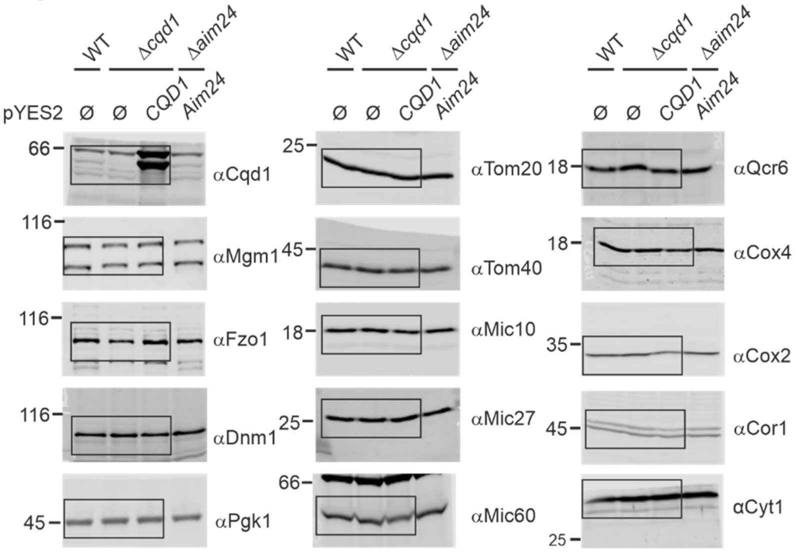

Fig. 7C

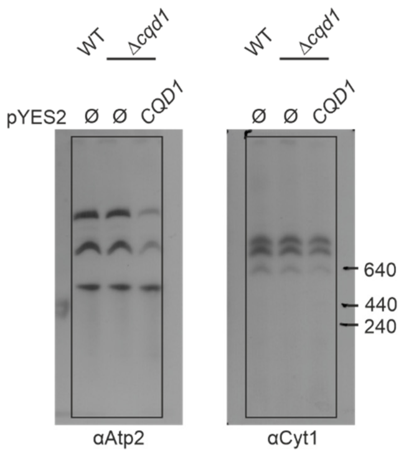

Journal of Cell Science • Supplementary information

Fig 8H

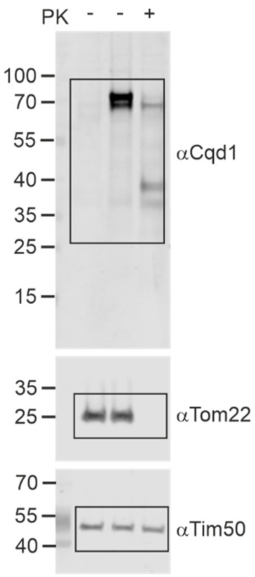

Fig 8I

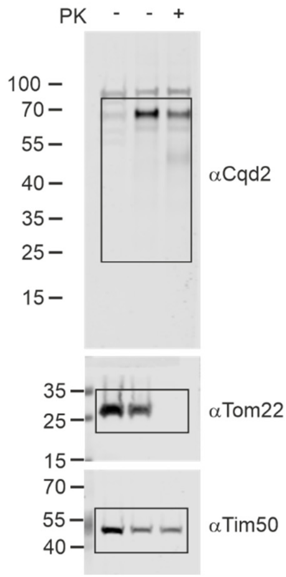

Fig. S1B

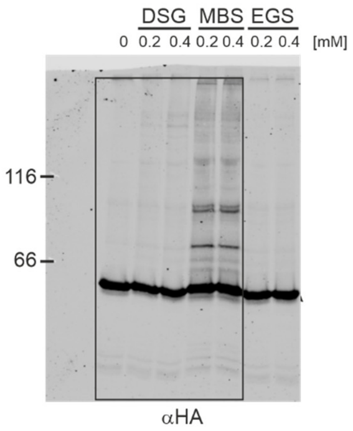

Fig. S4. Blot transparency

Table S1. Relative abundance of mitochondrial phospholipids.

Values of the graph shown in Figure 3. PC, phosphatidylcholine; PE, phosphatidylethanolamine; PS, phosphatidylserine; PI, phosphatidylinositol; CL, cardiolipin; PG, phosphatidylglycerol; PA, phosphatidic acid; MLCL, monolysocardiolipin.

average mol % lipid								
**Sample**	**PC**	**PE**	**PS**	**PI**	**PG**	**PA**	**CL**	**MLCL**
WT	36.17	27.30	5.05	16.48	0.15	4.17	10.45	0.22
Δ*ups1*	39.34	30.59	5.18	15.55	0.13	3.03	6.15	0.04
Δ*cqd1*	40.28	25.68	4.85	15.01	0.18	3.19	10.65	0.16
Δ*ups1* Δ*cqd1*	41.17	27.69	6.94	14.12	0.12	3.41	6.49	0.07
**Standard deviation**								
**Sample**	**PC**	**PE**	**PS**	**PI**	**PG**	**PA**	**CL**	**MLCL**
WT	5.29	3.20	0.66	4.15	0.04	0.48	1.41	0.11
Δ*ups1*	3.12	3.80	1.22	1.81	0.10	0.16	0.61	0.03
Δ*cqd1*	1.50	2.30	0.62	1.49	0.06	0.46	1.04	0.04
Δ*ups1* Δ*cqd1*	2.36	2.17	2.15	2.27	0.04	0.80	2.13	0.07

Table S2. Relative amount of different species of single phospholipids

Click here to download Table S2

Table S3. *S. cerevisiae* strains used in this study.

**Strain name**	**Genotype**	**Reference**
YPH499 (ρ^+)	*MATa ade2-101 his3-Δ200 leu2- trp1-Δ63 ura3-52 lys2-801*	(Sikorski and Hieter, 1989)
YPH499 (ρ^0)	*MATa ade2-101 his3-Δ200 leu2- trp1-Δ63 ura3-52 lys2-801*	Harner et al., 2014
Δ*cqd1*	YPH499 *cqd1Δ::HIS3MX6*	This study
Δ*cqd2*	YPH499 *cqd2Δ::HIS3MX6*	This study
Δ*ups1*	YPH499 *ups1Δ::kanMX4*	This study
Δ*ups1*	YPH499 *ups1Δ::LEU2 (K. lactis)*	This study
Δ*tam41*	YPH499 *tam41Δ::LEU2 (K. lactis)*	This study
Δ*pgs1*	YPH499 *pgs1Δ::LEU2 (K. lactis)*	This study
Δ*gep4*	YPH499 *gep4Δ::LEU2 (K. lactis)*	This study
Δ*crd1*	YPH499 *crdΔ::LEU2 (K. lactis)*	This study
Δ*taz1*	YPH499 *taz1Δ::LEU2 (K. lactis)*	This study
Δ*cld1*	YPH499 *cld1Δ::LEU2 (K. lactis)*	This study
Δ*ups2*	YPH499 *ups2Δ::LEU2 (K. lactis)*	This study
Δ*mdm35*	YPH499 *mdm35Δ::LEU2 (K. lactis)*	This study
Δ*psd1*	YPH499 *psd1Δ::LEU2 (K. lactis)*	This study

Journal of Cell Science • Supplementary information

Δ*por1*	YPH499 *por1Δ::kanMX4*	This study
Δ*om14*	YPH499 *om14Δ::LEU2 (K. lactis)*	This study
Δ*mgm1*	YPH499 *mgm1Δ:: kanMX4*	Harner et al., 2014
Δ*fzo1*	YPH499 *fzo1Δ::HIS3MX6*	This study
Δ*ups1* Δ*cqd1*	YPH499 *cqd1Δ::HIS3MX6 ups1Δ::LEU2 (K. lactis)*	This study
Δ*ups1* Δ*cqd1*	YPH499 *cqd1Δ::HIS3MX6 ups1Δ::kanMX4*	This study
Δ*tam41* Δ*cqd1*	YPH499 *cqd1Δ::HIS3MX6 tam41Δ::LEU2 (K. lactis)*	This study
Δ*pgs1* Δ*cqd1*	YPH499 *cqd1Δ::HIS3MX6 pgs1Δ::LEU2 (K. lactis)*	This study
Δ*gep4* Δ*cqd1*	YPH499 *cqd1Δ::HIS3MX6 gep4Δ::LEU2 (K. lactis)*	This study
Δ*crd1* Δ*cqd1*	YPH499 *cqd1Δ::HIS3MX6 crd1Δ::LEU2 (K. lactis)*	This study
Δ*taz1* Δ*cqd1*	YPH499 *cqd1Δ::HIS3MX6 taz1Δ::LEU2 (K. lactis)*	This study
Δ*cld1* Δ*cqd1*	YPH499 *cqd1Δ::HIS3MX6 cld1Δ::LEU2 (K. lactis)*	This study
Δ*ups2* Δ*cqd1*	YPH499 *cqd1Δ::HIS3MX6 ups2Δ::LEU2 (K. lactis)*	This study
Δ*mdm35* Δ*cqd1*	YPH499 *cqd1Δ::HIS3MX6 mdm35Δ::LEU2 (K. lactis)*	This study
Δ*psd1* Δ*cqd1*	YPH499 *cqd1Δ::HIS3MX6 psd1Δ::LEU2 (K. lactis)*	This study
Δ*cqd1* Δ*cqd2*	YPH499 *cqd2Δ::HIS3MX6 cqd1Δ::kanMX4*	This study
Δ*ups1* Δ*cqd2*	YPH499 *ups1Δ::kanMX6 cqd2Δ::hphNT1*	This study
Δ*ups1* Δ*cqd2* Δ*cqd1*	YPH499 *cqd2Δ::HIS3MX6 cqd1Δ::kanMX4 ups1Δ::LEU2 (K. lactis)*	This study
CQD1-3xHA	YPH499 *CQD1-3xHA::HIS3MX6*	This study
CQD1-3xMyc	YPH499 *CQD1::3xMyc::TRP1 (K. lactis)*	This study
POR1-3xHA	YPH499 *POR1-3xHA::HIS3MX6*	This study
OM14-3xHA	YPH499 *OM14-3xHA::HIS3MX6*	This study
OM14-3xHA Δ*por1*	YPH499 *OM14-3xHA::HIS3MX6 por1Δ:: LEU2 (K. lactis)*	This study
POR1-3xHA Δ*om14*	YPH499 *POR1-3xHA::TRP1 (K. lactis) HIS3MX6om14Δ::LEU2 (K. lactis)*	This study
Δ*mic60 OM14-3xHA*	YPH499 *mic60Δ::HIS3MX6 OM14-3xHA::TRP1 (K. lactis)*	This study
WT mKate	YPH499 *ho-PGK1pr-su9-KATE2::kanMX4*	This study
Δ*cqd1* mKate	YPH499 *cqd1Δ::HIS3MX6 ho-PGK1pr-su9-KATE2::kanMX4*	This study
Δ*ups1* mKate	YPH499 *ups1Δ::LEU2 (K. lactis) ho-PGK1pr-su9-KATE2::kanMX4*	This study
Δ*ups1* Δ*cqd1* mKate	YPH499 *ups1Δ::LEU2 (K. lactis) cqd1Δ::HIS3MX6 ho-PGK1pr-su9-KATE2::kanMX4*	This study
Δ*ups1 CQD1-3xHA*	YPH499 *cqd1Δ::CQD1-3xHA::HIS3MX6 ups1::LEU2 (K. lactis)*	This study
MIC10-3xHA	YPH499 *MIC10-3xHA::HIS3MX6*	(Harner et al., 2011)
MIC60-3xHA	YPH499 *MIC60-3xHA::kanMX4*	(Harner et al., 2011)

Journal of Cell Science • Supplementary information

Table S4. Primers used in this study.

Construct	Primer name	Sequence
pYES2-*CQD1*	*CQD1*(SacI)_for	CCCGAGCTCATGTCATTTTTAAAGTTCGC
	CQD1-int(EcoRI)_rev	CCCGAATTCACGTAATATAGGCAGCGAACTTTCA AATAAGTCCAAG
	CQD1-int(EcoRI)_for	CGTGAATTCGGGTTTAAGC
	CQD1(NotI)_rev	CCCGCGGCCGCTTAATAATTAGGACACAATTG
pRS316-*CQD1*	F HindIII *CQD1* (gib)	GCAGGAATTCGATATCAAGCTTGGTACTGGAAAG ATCGCGTTC
	R XhoI *CQD1*	CTTACCGGGCCCCCCCTCGACGTACCGTTGCCT TATTGTTC
pRS316-*cqd1(K275A)*	Cqd1_K275A_for	GCGATCTTGCATCCAAATGTAAG
	Cqd1_K275A_rev	GATGGCACACCAACGATTTC
pRS316-*cqd1(D288A)*	Cqd1_D288A_for	GCTTTGAAAATAATGAAATTCTG
	Cqd1_D288A_rev	TCTCCGGATCTGAGATCTTAC
pRS316- *cqd1(E330A)* pYES2-*cqd1(E330A)*	Cqd1_E330A_for	GCGGCGTTAAACCTGGAAAG
	Cqd1_E330A_rev	AATTCTTAGATCCAACTGAATA
pRS316-*CQD1-3xHA*	F HindIII *CQD1* (gib)	GCAGGAATTCGATATCAAGCTTGGTACTGGAAAG ATCGCGTTC
	Gib(XhoI)*ADH1*terR1	ACCGGGCCCCCCCTCGAGGTAGAGGTGTGGTCA ATAAG
pYES2-*CQD2*	*CQD2*(SacI)_for	CCCGAGCTCATGATGACCAAAGCTTTTTTTAAC
	CQD2(XhoI)_rev	CCCCTCGAGTTAAGATGACAACCAAGTCTTCG
pYX233-*CQD2*	pYXCQD2 (EcoRI)_for	CTACAAAAAACACATACAGGAATTCATGATGACC AAAGCTTTTTT
	pYXCQD2(NheI)_rev	ATTGTTCCTTATTCAGTTAGCTAGCTTAAGATGAC AACCAAGTC

Journal of Cell Science • Supplementary information

Table S5. Antibodies used in this study.

Antibody	Source	Identifier/Reference	Dilution
Mouse monoclonal anti-HA	Santa Cruz	Cat. #sc-7392	1:250
Mouse monoclonal anti-Pgk1	Life Technologies	Cat. #459250	1:250
Mouse monoclonal anti-Pgk1	MPI of Biochemistry	(Izawa et al., 2017)	1:250
Rabbit polyclonal anti-Cqd1	LMU Munich	This study	1:25
Rabbit polyclonal anti-Cqd2	LMU Munich	This study	1:25
Rabbit polyclonal anti-Tim17	LMU Munich	(Moro et al., 1999)	1:500
Rabbit polyclonal anti-Tim23	LMU Munich	(Moro et al., 1999)	1:500
Rabbit polyclonal anti-Tim50	LMU Munich	(Mokranjac et al., 2003)	1:500
Rabbit polyclonal anti-Tom20	LMU Munich	(Krimmer et al., 2001)	1:500
Rabbit polyclonal anti-Tom22	LMU Munich	(Krimmer et al., 2001)	1:500
Rabbit polyclonal anti-Tom40	LMU Munich	(Kiebler et al., 1990)	1:1,000
Rabbit polyclonal anti-Tom70	LMU Munich	(Schlossmann et al., 1996)	1:500
Rabbit polyclonal anti-Hep1	LMU Munich	(Sichting et al., 2005)	1:250
Rabbit polyclonal anti-Atp2	LMU Munich	(Izawa et al., 2017)	1:1,000
Rabbit polyclonal anti-Fzo1	LMU Munich	(Basch et al., 2020)	1:250
Rabbit polyclonal anti-Ugo1	LMU Munich	(Basch et al., 2020)	1:250
Rabbit polyclonal anti-Mgm1	LMU Munich	(Harner et al., 2016)	1:250
Rabbit polyclonal anti-Dnm1	LMU Munich	(Harner et al., 2016)	1:250
Rabbit polyclonal anti-Mdj1	LMU Munich	(Rowley et al., 1994)	1:250
Rabbit polyclonal anti-Mic10	LMU Munich	(Harner et al., 2011)	1:250
Rabbit polyclonal anti-Mic19	LMU Munich	(Harner et al., 2011)	1:250
Rabbit polyclonal anti-Mic26	LMU Munich	(Harner et al., 2011)	1:250
Rabbit polyclonal anti-Mic27	LMU Munich	(Harner et al., 2011)	1:250
Rabbit polyclonal anti-Mic60	LMU Munich	(Harner et al., 2011)	1:250
Rabbit polyclonal anti-Por1	LMU Munich	(Krimmer et al., 2001)	1:1,000
Rabbit polyclonal anti-Om45	LMU Munich	(Waizenegger et al., 2003)	1:250
Rabbit polyclonal anti-Rip1	LMU Munich	(Cruciat et al., 1999)	1:250
Rabbit polyclonal anti-Cor1	LMU Munich	(Cruciat et al., 2000)	1:250
Rabbit polyclonal anti-Cor2	LMU Munich	(Vogel et al., 2006)	1:250
Rabbit polyclonal anti-Cyt1	LMU Munich	(Izawa et al., 2017)	1:250
Rabbit polyclonal anti-Cox2	LMU Munich	(Izawa et al., 2017)	1:250
Rabbit polyclonal anti-Cox4	LMU Munich	(Cruciat et al., 2000)	1:250
Rabbit polyclonal anti-Qcr6	LMU Munich	(Cruciat et al., 1999)	1:250
IRDye 680RD Goat anti-Mouse IgG	LI-COR	Cat. #962-68070	1:10,000
IRDye 800CW Goat anti-Rabbit IgG	LI-COR	Cat. #926-32211	1:10,000
Goat Anti-Rabbit IgG-HRP Conjugate	BIO-RAD	Cat. #170-6515	1:10,000

Journal of Cell Science • Supplementary information

Supplementary References

Basch, M., Wagner, M., Rolland, S., Carbonell, A., Zeng, R., Khosravi, S., Schmidt, A., Aftab, W., Imhof, A., Wagener, J.*, et al.* (2020). Msp1 cooperates with the proteasome for extraction of arrested mitochondrial import intermediates. Mol Biol Cell *31*, 753-767.

Cruciat, C.M., Brunner, S., Baumann, F., Neupert, W., and Stuart, R.A. (2000). The cytochrome bc1 and cytochrome c oxidase complexes associate to form a single supracomplex in yeast mitochondria. J Biol Chem *275*, 18093-18098.

Cruciat, C.M., Hell, K., Folsch, H., Neupert, W., and Stuart, R.A. (1999). Bcs1p, an AAA-family member, is a chaperone for the assembly of the cytochrome bc(1) complex. EMBO J *18*, 5226-5233.

Harner, M., Korner, C., Walther, D., Mokranjac, D., Kaesmacher, J., Welsch, U., Griffith, J., Mann, M., Reggiori, F., and Neupert, W. (2011). The mitochondrial contact site complex, a determinant of mitochondrial architecture. EMBO J *30*, 4356-4370.

Harner, M.E., Unger, A.K., Geerts, W.J., Mari, M., Izawa, T., Stenger, M., Geimer, S., Reggiori, F., Westermann, B., and Neupert, W. (2016). An evidence based hypothesis on the existence of two pathways of mitochondrial crista formation. Elife *5*.

Izawa, T., Park, S.H., Zhao, L., Hartl, F.U., and Neupert, W. (2017). Cytosolic Protein Vms1 Links Ribosome Quality Control to Mitochondrial and Cellular Homeostasis. Cell *171*, 890-903 e818.

Kiebler, M., Pfaller, R., Sollner, T., Griffiths, G., Horstmann, H., Pfanner, N., and Neupert, W. (1990). Identification of a mitochondrial receptor complex required for recognition and membrane insertion of precursor proteins. Nature *348*, 610-616.

Krimmer, T., Rapaport, D., Ryan, M.T., Meisinger, C., Kassenbrock, C.K., Blachly-Dyson, E., Forte, M., Douglas, M.G., Neupert, W., Nargang, F.E.*, et al.* (2001). Biogenesis of porin of the outer mitochondrial membrane involves an import pathway via receptors and the general import pore of the TOM complex. J Cell Biol *152*, 289-300.

Mokranjac, D., Paschen, S.A., Kozany, C., Prokisch, H., Hoppins, S.C., Nargang, F.E., Neupert, W., and Hell, K. (2003). Tim50, a novel component of the TIM23 preprotein translocase of mitochondria. EMBO J *22*, 816-825.

Moro, F., Sirrenberg, C., Schneider, H.C., Neupert, W., and Brunner, M. (1999). The TIM17.23 preprotein translocase of mitochondria: composition and function in protein transport into the matrix. EMBO J *18*, 3667-3675.

Rowley, N., Prip-Buus, C., Westermann, B., Brown, C., Schwarz, E., Barrell, B., and Neupert, W. (1994). Mdj1p, a novel chaperone of the DnaJ family, is involved in mitochondrial biogenesis and protein folding. Cell *77*, 249-259.

Schlossmann, J., Lill, R., Neupert, W., and Court, D.A. (1996). Tom71, a novel homologue of the mitochondrial preprotein receptor Tom70. J Biol Chem *271*, 17890-17895.

Sichting, M., Mokranjac, D., Azem, A., Neupert, W., and Hell, K. (2005). Maintenance of structure and function of mitochondrial Hsp70 chaperones requires the chaperone Hep1. Embo J *24*, 1046-1056.

Sikorski, R.S., and Hieter, P. (1989). A system of shuttle vectors and yeast host strains designed for efficient manipulation of DNA in Saccharomyces cerevisiae. Genetics *122*, 19-27.

Vogel, F., Bornhovd, C., Neupert, W., and Reichert, A.S. (2006). Dynamic subcompartmentalization of the mitochondrial inner membrane. J Cell Biol *175*, 237-247.

Waizenegger, T., Stan, T., Neupert, W., and Rapaport, D. (2003). Signal-anchor domains of proteins of the outer membrane of mitochondria: structural and functional characteristics. J Biol Chem *278*, 42064-42071.

Journal of Cell Science • Supplementary information
