# Supplementary material for: The UbiB family member Cqd1 forms a novel membrane contact site in mitochondria
Source: J Cell Sci. 2023 May 18;136(10):jcs260578. doi: 10.1242/jcs.260578 (PMC10214851; doi:10.1242/jcs.260578)
Supplement: Supplementary file 1 [file joces-136-260578_review_history.pdf]

The UbiB family member Cqd1 forms a novel membrane contact site in mitochondria

Siavash Khosravi, Xenia Chelius, Ann-Katrin Unger, Daniela Rieger, Johanna Frickel, Timo Sachsenheimer, Christian Lüchtenborg, Rico Schieweck, Britta Brügger, Benedikt Westermann, Till Klecker, Walter Neupert and Max E. Harner
DOI: 10.1242/jcs.260578

Editor: Jennifer Lippincott-Schwartz

Review timeline
Original submission: 28 August 2022
Editorial decision: 17 October 2022
First revision received: 17 February 2023
Accepted: 11 April 2023

Original submission

First decision letter

MS ID#: JOCES/2022/260578

MS TITLE: The UbiB family member Cqd1 forms a novel membrane contact site in mitochondria

AUTHORS: Siavash Khosravi, Xenia Chelius, Ann-Katrin Unger, Johanna Frickel, Timo Sachsenheimer, Christian Luechtenborg, Rico Schieweck, Britta Bruegger, Benedikt Westermann, Till Klecker, Walter Neupert, and Max E Harner
ARTICLE TYPE: Research Article

We have now reached a decision on the above manuscript.

To see the reviewers' reports and a copy of this decision letter, please go to: https://submit-jcs.biologists.org and click on the 'Manuscripts with Decisions' queue in the Author Area. (Corresponding author only has access to reviews.)

As you will see, the reviewers raise a number of substantial criticisms that prevent me from accepting the paper at this stage. They suggest, however, that a revised version might prove acceptable, if you can address their concerns. If you think that you can deal satisfactorily with the criticisms on revision, I would be pleased to see a revised manuscript. We would then return it to the reviewers.

Please ensure that you clearly highlight all changes made in the revised manuscript. Please avoid using 'Tracked changes' in Word files as these are lost in PDF conversion.

I should be grateful if you would also provide a point-by-point response detailing how you have dealt with the points raised by the reviewers in the 'Response to Reviewers' box. Please attend to all of the reviewers' comments. If you do not agree with any of their criticisms or suggestions please explain clearly why this is so.

Reviewer 1

Advance summary and potential significance to field

© 2023. Published by The Company of Biologists under the terms of the Creative Commons Attribution License (https://creativecommons.org/licenses/by/4.0/).

In the manuscript by Khosravi et al., the authors characterize the functional role in yeast of the conserved UbiB family member and mitochondrial inner membrane protein Cqd1. The authors identify a potential role for the protein in phospholipid metabolism, finding a negative genetic interaction between Cqd1 and the PA transporter Ups1 that is not rescued by a mutation in the kinase-like domain of Cqd1. In support of a role in phospholipid metabolism, the authors identify a defect in mitochondrial PA levels in the absence of Cqd1. The authors further identify that combined loss of Cqd1 and Ups1 leads to synthetic defects in mitochondrial import and mitochondrial morphology, likely due to loss of mitochondrial fusion via impaired processing of Mgm1. Recent work from the Pagliarini lab suggests that Cqd1 plays a role in coenzyme Q metabolism and is antagonized by the function of another UbiB family member, Cqd2. Building on this work, the authors find that this antagonistic relationship extends to the synthetic growth defect of Cqd1 and Ups1. The authors also explore the effect of overexpression of Cqd1, showing that mitochondrial morphology and cell growth are negatively impacted, possibly due to defects in ATP synthase dimerization.
Finally, in addition to exploring a role for Cqd1 in phospholipid metabolism, the authors provide evidence that Cqd1 plays a role in bridging the inner and outer membranes. The authors use an established biochemical assay to suggest that Cqd1 fractionates with IM/OM contact site proteins, self-interacts in a ~400 kDa complex, and physically interacts with the outer membrane proteins Om14 and Por1.
In total, the work of the authors is generally carefully performed and there are several interesting observations in the manuscript. The work reveals a role for Cqd1 in phospholipid metabolism, confirms a general antagonistic relationship between Cqd1 and Cqd2, and identifies a potential role for Cqd1 as a bridging protein between the inner and outer membranes.

Comments for the author

The work suggesting Cqd1 is a contact site protein is not convincing as shown and the paper would be greatly strengthened by evaluating whether the interaction between Cqd1 and Om14/Por1 is relevant to Cqd1 function in phospholipid metabolism.

Major points:
1. While the authors identify a physical interaction between Cqd1 and the outer membrane proteins Om14 and Por1, they do not evaluate whether these interactions are functionally meaningful. Does the role of Cqd1 in phospholipid metabolism depended on its interaction with Om14 or Por1? Do the proteins share common genetic interactions?

2. It is difficult to assess whether Cqd1 is a contact site protein based on the fractionation experiment in Fig. 6A as this assay does not appear particularly sensitive. Mic27 and Tim17 appear to have nearly identical profiles in this assay.
Additionally, as the authors note, TIM and TOM exist in a supercomplex. How does this profile compare to other inner membrane proteins such as respiratory components or other outer membrane proteins? Do Om14 and Por1 also have a similar distribution?

3. While it is interesting that overexpression of Cqd1 impacts mitochondrial morphology, the functional meaning of these experiments are difficult to interpret with relation to the authors' other findings. For example, are the phenotypes related to effects on phospholipid metabolism such as overproduction of PA? Are the same defects are observed if the Cqd1(E330A) mutant or any other inner membrane protein is overexpressed. Does co-overexpression of Cqd2 prevent the morphology phenotypes? Are Om14 or Por1 required for the phenotype?

Minor points:
1. Severe fragmentation of mitochondria in mutants that cannot process Mgm1 frequently leads to loss of mitochondrial genomes. The severe synthetic growth defect of Cqd1/Ups1 could in part be explained by a failure to maintain mitochondrial genomes. Can the authors clarify whether the growth defect of strains on synthetic glucose media is similar to rho0 cells or other cells that cannot undergo mitochondrial fusion (Fzo1 or Mgm1 deletion, for example?).
2. Can the authors comment on the lower band that is absent in the Cqd1/Ups1 double deletion in the Cyt1 blot in Figure 4B? There does not appear to be a defect in supercomplex assembly, but does the lower band represent Complex III monomers?

© 2023. Published by The Company of Biologists under the terms of the Creative Commons Attribution License (https://creativecommons.org/licenses/by/4.0/).

Reviewer 2

Advance summary and potential significance to field

Harner and colleagues describe in their manuscript a novel contact site protein, which seems to bridge inner and outer mitochondrial membrane. This protein has been previously linked to the coenzyme Q biosynthesis pathway, yet has a conserved kinase domain. The authors show here that Cqd1 is anchored to the inner membrane with its kinase domain exposed to the intermembrane space, that it interacts with Por1 and Om45, two outer membrane proteins, and thus forms a MICOS independent contact site and that its overexpression results in massively changed morphology and enhanced ER-mitochondrial clustering. Deletion of Cqd1 has mild effects on growth and mitochondrial function, though these seem to be enhanced if combined with deletions involved in lipid transfer such as psd1 or crd1 or ups1. In agreement, the mitochondrial lipidome has deficiencies in PA levels, suggesting that Cqd1 mediated contacts contribute to efficient lipid distribution.

Overall, this is a very complete study, where the authors describe the Cqd1 protein and its physiology in much detail. Their data clearly shows that Cqd1 has an additional role in the generation of a contact site between the inner membrane and the outer membrane. Even though the precise function is not yet clear, it is an important advance in our understanding of mitochondrial morphology. I have only a few comments and suggestions:

1. The authors describe in Figure 7D the massive alteration of the mitochondrial morphology due to the Cqd1 overexpression. As Cqd1 binds to Por1 and OM45. Both proteins have been linked to ER-mitochondrial contact sites. I am thus wondering if the effect of overexpression on the ER-recruitment may be an indirect effect as Por1 and/or OM45 may become clustered. Do the authors see the same phenotype if they do the overexpression in por1 or om45 deletion cells? This may become apparent already at the fluorescent microscopy analysis.

2. Along the same line, it would be helpful to have a higher resolution of the inner membrane structures.

3. How does the mitochondrial morphology look like if cqd1 and ups1 are deleted? Is this like wild-type?

4. Do the authors have any evidence, where Cqd1 binds to Por1 or OM45? Is the contact maintained if either protein is deleted? This may already indicate, which of the two is the main interactor. It is also possible to map the putative interface with AlphaFold2, which may be useful.

5. The authors suggest that mutations in the kinase domain destabilize the protein. Do they have evidence that the protein has kinase activity or is this an inactive kinase? This could be tested either with the purified intramembrane domain or by comparing the phosphoproteome of wild-type vs. cqd1 deletion cells. These experiments may go beyond the initial characterization, and could be part of a new study.

6. The authors should summarize their suggestions in a working model. It helps readers to follow their thoughts.

Comments for the author

see above

Reviewer 3

Advance summary and potential significance to field

In their work, Khosravi et al. characterize the function of the Cqd1 protein ultimately uncovering its unique contribution to mitochondrial ultrastructure.
This study was motivated by the homology of Cqd1 with Coq8/ADCK3 and the bacterial UbiB proteins, which are involved in coenzyme Q synthesis. Additionally, it follows a report by Kemmerer

© 2023. Published by The Company of Biologists under the terms of the Creative Commons Attribution License (https://creativecommons.org/licenses/by/4.0/).

et al. describing the role of Cqd1 and Cqd2 in coenzyme Q distribution between the inner mitochondrial and plasma membranes.
Traditional biochemical and growth analyses extend findings from previous high-throughput proteomic and genetic studies: Cqd1 is localized on the inner mitochondrial membrane, and negative epistasis is observed between Cqd1 and Ups1 which is involved in the import of the phosphatidic acid phospholipid precursor.
Independently of Ups1, Cqd1 deletion also shows a decrease in phosphatidic acid level. Although the Cqd1 and Ups1 double deletion strain surprisingly does not show a synthetic phenotype in terms of mitochondrial phospholipid composition, it is clear that the deletion of Cqd1 exacerbates mitochondrial protein import and protein processing defects observed in the Ups1 deletion strain. These defects are rescued by additional deletion of Cqd2, which opposes Cqd1-mediated coenzyme Q re-distribution to the plasma membrane as reported by Kemmerer et al. Further analysis of the biochemical context of Cqd1 interestingly reveals that Cqd1 localizes to a MICOS-independent contact site between the two mitochondrial membranes, interacting with Om14 and Por1 on the outer mitochondrial membrane. The role of Cqd1 in mitochondrial membrane organization is corroborated by the gross perturbation of mitochondrial morphology and ultrastructure upon Cqd1 overexpression.

Comments for the author

Although this work follows the discovery of the roles of Cqd1 and Cqd2 in coenzyme Q distribution, I believe it is of sufficient novelty for publication in JCS given the determination of Cqd1 context in the mitochondrial membrane, and characterization of the Cqd1 overexpression strain. I recommend this manuscript for publication in general, however I believe that the conclusions drawn from key Figures 6 and 7 showing the biochemical context of Cqd1 could be strengthened by a few additional experiments, detailed below.

1. There is compelling evidence that the activity of Cqd1 and Cqd2 is coordinated which suggest they may also interact with one another. The authors show the Cqd1-containing complex is approx. 400 kDa, which could accommodate Cqd2 as well as Cqd1, Om14 and Por1. Testing this possibility by generating a double tagged Cqd1 and Cqd2 strain would be a worthwhile and appropriate addition to this work.

2. The interaction of Om14 with Por1 seems substochiometric compared to the interaction of Om14 with Cqd1 based on Figure 6G. It would therefore be pertinent to confirm Om14 interaction in the Por1 IP shown in Figure 6H by western blot.
Similarly, western blotting of the cross-linked product from Figure 6F for Por1 and Om14 could strengthen the author’s hypothesis that the crosslinked products are indeed due to Cqd1 interaction with these two proteins.

3. The authors find that properly localized endogenous Cqd1 is processed (Figure 1B). It is interesting that Cqd1 precursor accumulates in whole cell lysate when Cqd1 is overexpressed (Figure 7B). Given the importance of Cqd1 overexpression for the conclusions of this study, it is necessary to exclude that the observed effects are due to mislocalized Cqd1 precursor. Therefore, I recommend that the authors investigate the topology of both mature and precursor Cqd1 in the overexpression strain.

First revision

<u>Author response to reviewers' comments</u>

Point by point response

We are grateful for the very positive comments from all three reviewers:

- *"In total, the work of the authors is generally carefully performed and there are several interesting observations in the manuscript. The work reveals a role for Cqd1 in phospholipid metabolism, confirms a general antagonistic relationship between Cqd1 and Cqd2, and identifies a potential role for Cqd1 as a bridging protein between the*

© 2023. Published by The Company of Biologists under the terms of the Creative Commons Attribution License (https://creativecommons.org/licenses/by/4.0/).

inner and outer membranes." (Reviewer #1)

- *"Overall, this is a very complete study, where the authors describe the Cqd1 protein and its physiology in much detail. Their data clearly shows that Cqd1 has an additional role in the generation of a contact site between the inner membrane and the outer membrane. Even though the precise function is not yet clear, it is an important advance in our understanding of mitochondrial morphology."* (Reviewer #2)
- *"Although this work follows the discovery of the roles of Cqd1 and Cqd2 in coenzyme Q distribution, I believe it is of sufficient novelty for publication in JCS given the determination of Cqd1 context in the mitochondrial membrane, and characterization of the Cqd1 overexpression strain."* (Reviewer #3)

We thank all reviewers for their constructive criticism and useful suggestions that helped us to improve the manuscript, as outlined below.

Reviewer 1

Major points:

1. While the authors identify a physical interaction between Cqd1 and the outer membrane proteins Om14 and Por1, they do not evaluate whether these interactions are functionally meaningful. Does the role of Cqd1 in phospholipid metabolism depended on its interaction with Om14 or Por1? Do the proteins share common genetic interactions?

We thank the reviewer for bringing up this point. *CQD1* and *POR1* share 19 genetic interactors. Interestingly, these include the ERMES subunits *GEM1* and *MMM1* (Kornmann et al., 2009; Kornmann et al., 2011; Stroud et al., 2011) and *MDM35* and *PSD1*. All these proteins are involved in the transport of phospholipids between the ER and mitochondria or the mitochondrial phospholipid metabolism (Clancey et al., 1993; Kannan et al., 2015; Kojima et al., 2019; Miyata et al., 2016; Potting et al., 2010; Trotter et al., 1993; Watanabe et al., 2015; Yu et al., 2015). This suggests that the physical interaction between Cqd1 and the Por1-Om14 complex is indeed functionally meaningful. Therefore, we added this interesting point in the discussion.

2. It is difficult to assess whether Cqd1 is a contact site protein based on the fractionation experiment in Fig. 6A as this assay does not appear particularly sensitive. Mic27 and Tim17 appear to have nearly identical profiles in this assay. Additionally, as the authors note, TIM and TOM exist in a supercomplex. How does this profile compare to other inner membrane proteins such as respiratory components or other outer membrane proteins? Do Om14 and Por1 also have a similar distribution?

We used this assay before to identify the MICOS complex (Harner et al., 2011a), one of the major contact site forming complexes. In this assay mild sonication is applied to generate mitochondrial membrane vesicles. The mitochondrial outer and inner membranes differ in their density allowing the subsequent vesicle separation through sucrose buoyant density gradient centrifugation. Outer membrane vesicles will be enriched in fractions of low density (here Fraction No. 4) whereas inner membrane vesicles will accumulate in fractions of high density (here Fraction No. 17). However, at contact sites, vesicles consisting of outer and inner membrane will be formed resulting in an enrichment of inner membrane proteins in fractions of intermediate density (here Fraction No. 10-14). As a proof of principle, we could show before that inhibition of contact site formation leads to a significant shift of the distribution of MICOS subunits from fractions of intermediate density to fractions of high density (Fig. 1, Fig. 4 and Supplemental Fig. S4A in Harner et al., 2011a).
In contrast to Tim17, Mic27 and Cqd1 show a significant protein amount in fractions of intermediate density in the experiments presented here, indicating that Cqd1 might be present in contact sites. We now explained this in more detail in the main text and highlighted the different distributions of the outer membrane protein Tom40, the inner membrane protein Tim17 as well as the (potential) contact site proteins Mic27 and Cqd1 through additional statistical analysis (Suppl Fig.1A). Importantly, we could show that Cqd1 is present in the inner membrane (Fig. 1 B,C) and that it interacts with Por1 and Om14 in the outer membrane (Fig. 6 F-J) proving the formation of a contact site. Unfortunately, we cannot decorate for additional proteins in the experiments shown in Fig. 6A, but we published the distribution of Por1, Om45, Cox2 and Cor2 before (Harner et al., 2011a; Fig. 1 and 4). These data also show that there is a clear difference between the distribution of

© 2023. Published by The Company of Biologists under the terms of the Creative Commons Attribution License (https://creativecommons.org/licenses/by/4.0/).

MICOS subunits/contact site proteins and other inner membrane proteins like Cox2, Cor2 (respiratory components), Tim17 and Tim23.

The TIM23-TOM supercomplex indeed forms a contact site between the mitochondrial inner and outer membranes. However, this contact site is formed only transiently during protein import. A permanent contact can only be induced when translocation intermediates are arrested by fusion of precursor proteins to tightly folded domains, which cannot pass the TOM complex, such as GFP, IgGs, or DHFR (Chacinska et al., 2003; Chacinska et al., 2010; Harner et al., 2011b; Schleyer and Neupert, 1985; Schwaiger et al., 1987). Thus, in the absence of precursor proteins, conditions that were applied here, subunits of the TIM23 and TOM complexes behave like classical inner or outer membrane proteins. We now added the missing information in the introduction.

3. While it is interesting that overexpression of Cqd1 impacts mitochondrial morphology, the functional meaning of these experiments are difficult to interpret with relation to the authors' other findings. For example, are the phenotypes related to effects on phospholipid metabolism such as overproduction of PA? Are the same defects are observed if the Cqd1(E330A) mutant or any other inner membrane protein is overexpressed. Does co-overexpression of Cqd2 prevent the morphology phenotypes? Are Om14 or Por1 required for the phenotype?

We thank all three reviewers for bringing up this important point. We extended our analysis of *CQD1* overexpression and included the *cqd1(E330A)* allele, *CQD2*, Δ*om14* and Δ*por1*. We were already quite cautious in the interpretation of the overexpression results in the original manuscript. Our new data revealed that at least some of the overexpressed protein ends up in the outer membrane where it apparently adopts a new function, which is independent of Om14 or Por1. The physiological significance of the mitochondrial ER interactions is currently unclear. However, we feel that these results are important as they help to understand the previously reported finding that *CQD2* overexpression suppresses the phenotypes of ERMES mutants (Tan et al. 2013). We present our new results in a new paragraph and a new Figure 8 and we also updated the discussion. Furthermore, we analyzed the phenotype caused by overexpression of the third UbiB protein kinase- like family member Coq8 on yeast cell growth and mitochondrial morphology. Although this did not result in a comparable growth phenotype, mitochondria tended to form aggregates (not shown).

Minor points:

1. Severe fragmentation of mitochondria in mutants that cannot process Mgm1 frequently leads to loss of mitochondrial genomes. The severe synthetic growth defect of Cqd1/Ups1 could in part be explained by a failure to maintain mitochondrial genomes. Can the authors clarify whether the growth defect of strains on synthetic glucose media is similar to rho0 cells or other cells that cannot undergo mitochondrial fusion (Fzo1 or Mgm1 deletion, for example?).

We thank the reviewer for this comment and tested whether ρ0 WT cells or cells lacking MGM1 or *FZO1* showed a growth phenotype comparable to Δ*cqd1* Δ*ups1* cells. None of the fusion-deficient strains showed a growth defect on SCD that was similarly severe as that of Δ*cqd1* Δ*ups1*. Moreover, the Δ*cqd1* Δ*ups1* double deletion mutant was still able to grow on respiratory medium, clearly demonstrating that it contains functional mtDNA. We added this control in Fig. 4.

2. Can the authors comment on the lower band that is absent in the Cqd1/Ups1 double deletion in the Cyt1 blot in Figure 4B? There does not appear to be a defect in supercomplex assembly, but does the lower band represent Complex III monomers?

This band probably represents a complex III dimer. It is unclear why the amount of complex III dimer in the Δ*cqd1* Δ*ups1* double deletion mutant was reduced in this particular experiment. We exchanged the experiment presented in Fig. 4B by a new BN-PAGE showing that there is no significant difference in formation of respiratory chain complexes.

© 2023. Published by The Company of Biologists under the terms of the Creative Commons Attribution License (https://creativecommons.org/licenses/by/4.0/).

Reviewer 2

1. The authors describe in Figure 7D the massive alteration of the mitochondrial morphology due to the Cqd1 overexpression. As Cqd1 binds to Por1 and OM45. Both proteins have been linked to ER- mitochondrial contact sites. I am thus wondering if the effect of overexpression on the ER-recruitment may be an indirect effect as Por1 and/or OM45 may become clustered. Do the authors see the same phenotype if they do the overexpression in por1 or om45 deletion cells? This may become apparent already at the fluorescent microscopy analysis.

We thank reviewer 2 for this important comment, that was also addressed by reviewer 1. Please see our response to major point 3 of reviewer 1.

2. Along the same line, it would be helpful to have a higher resolution of the inner membrane structures.

Unfortunately, it is technically not possible to obtain a higher resolution. The resolution is limited by the thickness of the ultrathin sections used for electron microscopy, which is about 60 nm in the z axis. Membrane structures that cross the section not perpendicularly may appear fuzzy in 2D images. We feel that the quality of the electron micrographs compares very well to other images of yeast mitochondrial ultrastructure that can be found in the literature.

3. How does the mitochondrial morphology look like if cqd1 and ups1 are deleted? Is this like wild- type?

Deletion of *CQD1* does not result in altered mitochondrial morphology. Deletion of *UPS1*, however, leads to partial fragmentation of mitochondria and the simultaneous deletion of both genes causes virtually complete fragmentation of the mitochondrial network (Fig. 4E). Interestingly, analysis of mitochondrial architecture does not reflect the morphological phenotype. Previous studies showed that the mitochondrial architecture of the Δ*ups1* single deletion is wild typic (Connerth et al., 2012) like that of the Δ*cqd1* single deletion (Fig. 7D and Suppl. Fig. 2). Although the double deletion mutant Δ*cqd1* Δ*ups1* showed a dramatic morphological phenotype, initial EM analysis revealed that mitochondrial architecture is not altered. Since these are preliminary results with a minor impact on our conclusions we would prefer not to include these data in the manuscript.

4. Do the authors have any evidence, where Cqd1 binds to Por1 or OM45? Is the contact maintained if either protein is deleted? This may already indicate, which of the two is the main interactor. It is also possible to map the putative interface with AlphaFold2, which may be useful.

The reviewer is addressing an important question here. We generated the necessary new yeast mutants Δ*om14* Por1-3xHA and Δ*por1* Om14-3xHA to identify the primary interactor of Cqd1. Immunoprecipitation revealed that it is still possible to co-IP Cqd1 with Om14-3xHA and Por1-3xHA although Por1 or Om14 were absent, indicating that Cqd1 interacts directly with both proteins, possibly in an independent manner. It should be noted that Cqd1 might also interact with Por2, the paralog of Por1 which is still present in Δ*por1*. We added these new data in Fig. 6.

5. The authors suggest that mutations in the kinase domain destabilize the protein. Do they have evidence that the protein has kinase activity or is this an inactive kinase? This could be tested either with the purified intramembrane domain or by comparing the phosphoproteome of wild-type vs. cqd1 deletion cells. These experiments may go beyond the initial characterization, and could be part of a new study.

We could not manage to recombinantly express soluble Cqd1. Therefore, we could not test for kinase activity. We agree with the reviewer that analysis of the phosphoproteome is beyond the scope of this study.

6. The authors should summarize their suggestions in a working model. It helps readers to follow their thoughts.

© 2023. Published by The Company of Biologists under the terms of the Creative Commons Attribution License (https://creativecommons.org/licenses/by/4.0/).

We prefer to draw our conclusions in the discussion and hope that the amendments we made to the text of the revised version make it more comprehensible to the reader.

Reviewer 3

1. There is compelling evidence that the activity of Cqd1 and Cqd2 is coordinated, which suggest they may also interact with one another. The authors show the Cqd1-containing complex is approx. 400 kDa, which could accommodate Cqd2 as well as Cqd1, Om14 and Por1. Testing this possibility by generating a double tagged Cqd1 and Cqd2 strain would be a worthwhile and appropriate addition to this work.

We also thank reviewer 3 for her or his important suggestions. Indeed, it is well possible that Cqd1 and Cqd2 form a complex in the mitochondrial inner membrane. To test this, we followed the reviewer's suggestion and analyzed the integration of Cqd2 in a high molecular weight complex by BN-PAGE (Rebuttal Fig. 1). Although we could show that Cqd1-3xHA is present in a 400 kDa complex, we could not get a signal for Cqd2-3xHA. This might either suggest that Cqd2 is not present in the Cqd1 containing complex or that the epitope is not accessible for the antibody. In addition, we tested whether Cqd1 co-immunoprecipitates with Cqd2-3xHA. However, Cqd2-3xHA is highly unstable and therefore does not allow a reasonable co-IP. As these are negative observations, we prefer not to include these data into the manuscript.

[NOTE: We have removed unpublished data that had been provided for the referees in confidence.]

2. The interaction of Om14 with Por1 seems substochiometric compared to the interaction of Om14 with Cqd1 based on Figure 6G. It would therefore be pertinent to confirm Om14 interaction in the Por1 IP shown in Figure 6H by western blot. Similarly, western blotting of the cross-linked product from Figure 6F for Por1 and Om14 could strengthen the author's hypothesis that the crosslinked products are indeed due to Cqd1 interaction with these two proteins.

Unfortunately, we do not have an antibody against Om14, therefore we cannot decorate for this protein in the IP shown in Fig. 6H (now Fig. 6G). However, to further analyze the interactions of Cqd1 with Om14 and Por1, we followed the suggestion of reviewer 2 and found that Cqd1 interacts directly with both proteins independent of the presence of the other outer membrane interaction partner (reviewer 2, point 4). As the exact identity of the crosslinks remains unclear, we shifted this experiment to the supplement.

3. The authors find that properly localized endogenous Cqd1 is processed (Figure1B). It is interesting that Cqd1 precursor accumulates in whole cell lysate when Cqd1 is overexpressed (Figure 7B). Given the importance of Cqd1 overexpression for the conclusions of this study, it is necessary to exclude that the observed effects are due to mis-localized Cqd1 precursor. Therefore, I recommend that the authors investigate the topology of both mature and precursor Cqd1 in the overexpression strain.

We thank the reviewer for this suggestion. Indeed, we found that overexpression of *CQD1* as well as *CQD2* results in a significantly altered localization of these proteins in the mitochondrial outer membrane. At the same time, overexpression of both proteins results in virtually identical phenotypes, reduced cell growth and formation of mitochondria-ER aggregates. Interestingly, it was shown before, that overexpression of *CQD2* can rescue the phenotype caused by deletion of ERMES subunits (Tan et al., 2013). Therefore, we conclude that this so far unexplained rescue is caused by the tethering of mitochondria to the ER identified here. We added this novel important information in the main text and Fig. 8. Additionally, we adapted the text accordingly. Please see also our response to reviewer 1, major point 3, for this important issue.

Further point

Please note that the experiment shown in Fig. 1C has been replaced by a new experiment.

© 2023. Published by The Company of Biologists under the terms of the Creative Commons Attribution License (https://creativecommons.org/licenses/by/4.0/).

References

Chacinska, A., Rehling, P., Guiard, B., Frazier, A.E., Schulze-Specking, A., Pfanner, N., Voos, W., and Meisinger, C. (2003). Mitochondrial translocation contact sites: separation of dynamic and stabilizing elements in formation of a TOM-TIM-preprotein supercomplex. EMBO J *22*, 5370-5381.

Chacinska, A., van der Laan, M., Mehnert, C.S., Guiard, B., Mick, D.U., Hutu, D.P., Truscott, K.N., Wiedemann, N., Meisinger, C., Pfanner, N., *et al.* (2010). Distinct forms of mitochondrial TOM-TIM supercomplexes define signal-dependent states of preprotein sorting. Mol Cell Biol *30*, 307-318.

Clancey, C.J., Chang, S.C., and Dowhan, W. (1993). Cloning of a gene (PSD1) encoding phosphatidylserine decarboxylase from Saccharomyces cerevisiae by complementation of an Escherichia coli mutant. J Biol Chem *268*, 24580-24590.

Connerth, M., Tatsuta, T., Haag, M., Klecker, T., Westermann, B., and Langer, T. (2012). Intramitochondrial transport of phosphatidic acid in yeast by a lipid transfer protein. Science *338*, 815- 818.

Harner, M., Korner, C., Walther, D., Mokranjac, D., Kaesmacher, J., Welsch, U., Griffith, J., Mann, M., Reggiori, F., and Neupert, W. (2011a). The mitochondrial contact site complex, a determinant of mitochondrial architecture. EMBO J *30*, 4356-4370.

Harner, M., Neupert, W., and Deponte, M. (2011b). Lateral release of proteins from the TOM complex into the outer membrane of mitochondria. EMBO J *30*, 3232-3241.

Kannan, M., Riekhof, W.R., and Voelker, D.R. (2015). Transport of phosphatidylserine from the endoplasmic reticulum to the site of phosphatidylserine decarboxylase2 in yeast. Traffic *16*, 123-134.

Kojima, R., Kakimoto, Y., Furuta, S., Itoh, K., Sesaki, H., Endo, T., and Tamura, Y. (2019). Maintenance of Cardiolipin and Crista Structure Requires Cooperative Functions of Mitochondrial Dynamics and Phospholipid Transport. Cell Rep *26*, 518-528 e516.

Kornmann, B., Currie, E., Collins, S.R., Schuldiner, M., Nunnari, J., Weissman, J.S., and Walter, P. (2009). An ER-mitochondria tethering complex revealed by a synthetic biology screen. Science *325*, 477-481.

Kornmann, B., Osman, C., and Walter, P. (2011). The conserved GTPase Gem1 regulates endoplasmic reticulum-mitochondria connections. Proc Natl Acad Sci U S A *108*, 14151-14156.

Miyata, N., Watanabe, Y., Tamura, Y., Endo, T., and Kuge, O. (2016). Phosphatidylserine transport by Ups2-Mdm35 in respiration-active mitochondria. J Cell Biol *214*, 77-88.

Potting, C., Wilmes, C., Engmann, T., Osman, C., and Langer, T. (2010). Regulation of mitochondrial phospholipids by Ups1/PRELI-like proteins depends on proteolysis and Mdm35. EMBO J *29*, 2888-2898.

Schleyer, M., and Neupert, W. (1985). Transport of proteins into mitochondria: translocational intermediates spanning contact sites between outer and inner membranes. Cell *43*, 339-350.

Schwaiger, M., Herzog, V., and Neupert, W. (1987). Characterization of translocation contact sites involved in the import of mitochondrial proteins. J Cell Biol *105*, 235-246.

Stroud, D.A., Oeljeklaus, S., Wiese, S., Bohnert, M., Lewandrowski, U., Sickmann, A., Guiard, B., van der Laan, M., Warscheid, B., and Wiedemann, N. (2011). Composition and topology of the endoplasmic reticulum-mitochondria encounter structure. J Mol Biol *413*, 743-750.

Tan, T., Ozbalci, C., Brugger, B., Rapaport, D., and Dimmer, K.S. (2013). Mcp1 and Mcp2, two novel proteins involved in mitochondrial lipid homeostasis. J Cell Sci *126*, 3563-3574.

Trotter, P.J., Pedretti, J., and Voelker, D.R. (1993). Phosphatidylserine decarboxylase from Saccharomyces cerevisiae. Isolation of mutants, cloning of the gene, and creation of a null allele. J Biol Chem *268*, 21416-21424.

Watanabe, Y., Tamura, Y., Kawano, S., and Endo, T. (2015). Structural and mechanistic insights into phospholipid transfer by Ups1-Mdm35 in mitochondria. Nat Commun *6*, 7922.

Yu, F., He, F., Yao, H., Wang, C., Wang, J., Li, J., Qi, X., Xue, H., Ding, J., and Zhang, P. (2015). Structural basis of intramitochondrial phosphatidic acid transport mediated by Ups1-Mdm35 complex. EMBO Rep *16*, 813-823.

© 2023. Published by The Company of Biologists under the terms of the Creative Commons Attribution License (https://creativecommons.org/licenses/by/4.0/).

Second decision letter

MS ID#: JOCES/2022/260578

MS TITLE: The UbiB family member Cqd1 forms a novel membrane contact site in mitochondria

AUTHORS: Siavash Khosravi, Xenia Chelius, Ann-Katrin Unger, Daniela Rieger, Johanna Frickel, Timo Sachsenheimer, Christian Luechtenborg, Rico Schieweck, Britta Bruegger, Benedikt Westermann, Till Klecker, Walter Neupert, and Max E Harner
ARTICLE TYPE: Research Article

I am happy to tell you that your manuscript has been accepted for publication in Journal of Cell Science, pending standard ethics checks.

Reviewer 1

Advance summary and potential significance to field

In their original manuscript, the authors provided evidence that Cqd1 plays a role in phospholipid metabolism and identified protein-protein interactions between Cqd1 and the outer membrane proteins Om14 and Por1. The primary concern I raised was that there was no evidence that the physical interaction between Om14/Por1/Cqd1 plays a functional role related to Cqd1 involvement in phospholipid metabolism.

Additionally, I raised the point that the evidence suggesting Cqd1 is a contact site protein was unconvincing and not supported by orthogonal approaches. In their revision, the authors failed to experimentally address either of these concerns. The authors now provide evidence that overexpression of Cqd1, previously used to support the model that Cqd1 is involved in phospholipid metabolism, leads to mistargeting of the protein to the outer membrane where it causes phenotypes independently of Om14/Por1. Thus, the manuscript makes three independent points about Cqd1: (1) Cqd1 is involved in phospholipid metabolism, (2) it interacts with outer membrane proteins Om14 and Por1, and (3) overexpressed Cqd1 targets to the outer membrane which may or may not form the molecular basis for increased ER/mitochondrial contact. However, how each observation relates to the other remains underexplored in the manuscript.

Comments for the author

1. The shared common genetic interactors between Cqd1 and Por1 could form the basis for experimentation testing whether the two proteins play a functional role together, as could assays testing for genetic interactions between Cqd1 and Om14 or Por1 in cell growth or mitochondrial morphology assays. The lack of functional connection between Cqd1 and Om14/Por1 remains a weakness of the manuscript.

2. While the authors previously used fractionation profiles to identify the MICOS complex as an inner/outer membrane contact site protein, that study and others used orthogonal approaches to demonstrate the key role of the MICOS complex at the interface between the two membranes. As the authors mention in their rebuttal, they previously used controls such as mic60Δ to show a shift in the distribution of contact site proteins, though did not perform a similar control here. The authors have not yet tested whether Om14/Por1 have similar profiles as Cqd1. Likewise, does loss of Om14 and Por1 cause a shift in distribution of Cqd1 or vice versa, which would help substantiate the physical interaction they have observed.

3. While it is potentially interesting that mistargeting of Cqd1 to the outer membrane correlates with increased ER-mitochondrial contact, the authors have not tested whether the mistargeting itself is required for the ER contact phenotype.
If Cqd1 is artificially targeted to the outer membrane, is this sufficient to induce ER contact? What is the functional basis for this phenotype? Can this phenotype be used to generate additional hypotheses regarding Cqd1 native function or involvement in phospholipid metabolism?

© 2023. Published by The Company of Biologists under the terms of the Creative Commons Attribution License (https://creativecommons.org/licenses/by/4.0/).

Without addressing these points, the manuscript reads like a collection of orthogonal observations that may or may not be related. This concern would be mitigated if the authors could demonstrate that the Om14/Por1 interaction with Cqd1 related to the function of the endogenous protein, which has not yet been tested.

Reviewer 2

Advance summary and potential significance to field

The authors addressed all concerns sufficiently. I have no further comments.

Comments for the author

see above

Reviewer 3

Advance summary and potential significance to field

The authors have made a solid effort to address all of my comments, and I am happy to recommend the manuscript for publication in its current form.

Comments for the author

Although further work is necessary to account for the complete composition of the proposed 400 kDa contact site complex, the finding that Cqd1 is able to interact with Om14 and Por1 independently is a good first step. I commend the authors for an honest discussion of the mislocalization of Cqd1 upon overexpression, and its ‘gain of function’ as an ER-mitochondrial tether once on the OMM.

© 2023. Published by The Company of Biologists under the terms of the Creative Commons Attribution License (https://creativecommons.org/licenses/by/4.0/).
